# Engineered plants provide a photosynthetic platform for the production of diverse human milk oligosaccharides

**DOI:** 10.1038/s43016-024-00996-x

**Published:** 2024-06-13

**Authors:** Collin R. Barnum, Bruna Paviani, Garret Couture, Chad Masarweh, Ye Chen, Yu-Ping Huang, Kasey Markel, David A. Mills, Carlito B. Lebrilla, Daniela Barile, Minliang Yang, Patrick M. Shih

**Affiliations:** 1 Department of Plant and Microbial Biology, University of California, Berkeley, Berkeley, CA USA; 2 Department of Plant Biology, University of California, Davis, Davis, CA USA; 3 Feedstocks Division, Joint Bioenergy Institute, Emeryville, CA USA; 4 Department of Food Science and Technology, University of California, Davis, Davis, CA USA; 5 Foods for Health Institute, University of California, Davis, Davis, CA USA; 6 Department of Chemistry, University of California, Davis, Davis, CA USA; 7 Environmental Genomics and Systems Biology Division, Lawrence Berkeley National Laboratory, Berkeley, CA USA; 8 Department of Viticulture and Enology, University of California, Davis, Davis, CA USA; 9 Department of Food, Bioprocessing and Nutrition Sciences, North Carolina State University, Raleigh, NC USA; 10 Innovative Genomics Institute, University of California, Berkeley, Berkeley, CA USA

**Keywords:** Molecular engineering in plants, Dietary carbohydrates, Mass spectrometry

## Abstract

Human milk oligosaccharides (HMOs) are a diverse class of carbohydrates which support the health and development of infants. The vast health benefits of HMOs have made them a commercial target for microbial production; however, producing the approximately 200 structurally diverse HMOs at scale has proved difficult. Here we produce a diversity of HMOs by leveraging the robust carbohydrate anabolism of plants. This diversity includes high-value and complex HMOs, such as lacto-*N*-fucopentaose I. HMOs produced in transgenic plants provided strong bifidogenic properties, indicating their ability to serve as a prebiotic supplement with potential applications in adult and infant health. Technoeconomic analyses demonstrate that producing HMOs in plants provides a path to the large-scale production of specific HMOs at lower prices than microbial production platforms. Our work demonstrates the promise in leveraging plants for the low-cost and sustainable production of HMOs.

## Abstract (web summary)

Human milk oligosaccharides (HMOs) are essential carbohydrates for the early development of infants. In this study, the intrinsic carbohydrate anabolism of plants is leveraged to produce a variety of HMOs, highlighting the potential of this method for commercial applications.

## Main

Human milk is a complete and comprehensive food evolved to nourish and protect infants. A key component to the distinct bioactive properties of human milk is the presence of a wide diversity of human milk oligosaccharides (HMOs) which are well documented in establishing the nascent gut microbiota of infants to prevent diseases and ensure healthy development^1–4^. While 75% of infants are supplemented with or exclusively fed infant formula in the first 6 months of life, current infant formulas are either devoid of HMOs or only contain one to two of the ~200 HMOs found in human milk, limiting the health outcomes of formula-fed infants^3,5,6^. In addition to their use for infant health, HMOs are being studied for their beneficial roles in adult health as a prebiotic to improve intestinal barrier function, lower gastrointestinal inflammation and treat irritable bowel diseases^7–11^; however, the study of HMO benefits in adults has been limited to a small subset of HMOs. Currently, commercial HMO production relies on microbial fermentation but, to date, microbial fermentation is only able to commercially produce two to five simple HMOs of the ~200 HMOs found in human milk at a scale suitable to supplement food products^6,12,13^. While five simple HMOs constitute a large portion of HMO mass in human milk, diverse HMOs with a range of linkages and degrees of polymerization enable the growth of beneficial gut microbes which have preferences for specific HMOs^14,15^. Thus, there is a need to develop biological platforms to produce a wider diversity of HMOs found in human milk, enabling the supplementation of food products for both infants and adults.

**Fig. 1:**
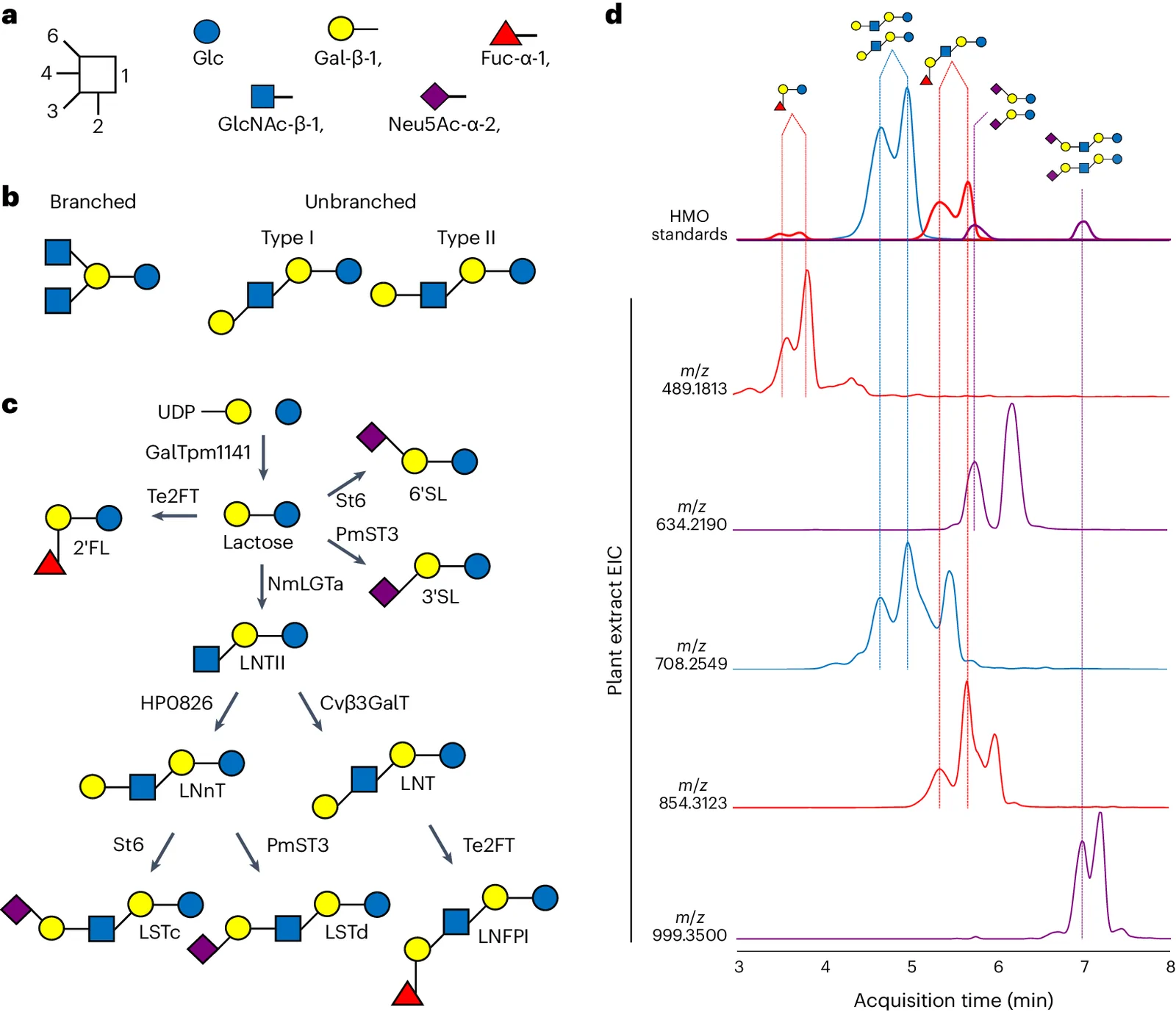
Production of all three HMO classes in planta. **a**, HMOs are composed of Glc, Gal, Fuc, GlcNAc and/or Neu5Ac connected via Gal-ß-1,3/4, GlcNAc-ß-1,3/6, Fuc-α-1,2/3/4 or Neu5Ac-α-2,3/6 glycosidic linkages. **b**, HMOs can be divided into branched, unbranched, type I and/or type II HMOs. **c**, HMO biosynthetic pathways used in this study for HMO production in planta. **d**, Extracted ion chromatograms (EIC) showing the identification of 2′FL, 3′SL, 6′SL, LNFPI, LSTa, LSTc and LNT/LNnT in extracts of individual plant leaves using LC–MS/MS (Q Exactive, Thermo Fisher Scientific). Red, blue and purple colouring denote fucosylated, neutral and acidic HMOs, respectively. Some further peaks are present due to in-source fragmentation of larger oligosaccharides with no available standards.

The combinatorial nature of glycosidic linkages, nucleotide sugar donors and oligosaccharide acceptor molecules enables the large diversity of HMOs found in human milk^16^. HMOs are composed of five distinct sugars—d-glucose (Glc), d-galactose (Gal), *N*-acetylglucosamine (GlcNAc), l-fucose (Fuc) and *N*-acetylneuraminic acid (Neu5Ac)—connected through various glycosidic linkages to generate a diverse range of molecular structures (Fig. 1a). HMO biosynthesis begins with the production of lactose which can be decorated with fucose or Neu5Ac to form a variety of trisaccharides and tetrasaccharides. Lactose can also be extended by glycosyltransferases through the addition of a GlcNAc-ß-1,3 or GlcNAc-ß-1,4 to form unbranched HMOs. Unbranched HMOs can be further extended by glycosyltransferases through the addition of a GlcNAc-ß-1,6 to produce branched HMOs (Fig. 1b). GlcNAc can undergo subsequent addition of Gal-ß-1,3 or Gal-ß-1,4 to form type I and type II HMOs, respectively (Fig. 1b). While HMOs can consist of all five monosaccharides, they are generally classified in three broad HMO groups on the basis of their composition: (1) neutral HMOs contain Glc, Gal and GlcNAc, (2) fucosylated HMOs contain a neutral core with one or more Fuc additions and (3) acidic HMOs contain a neutral core with one or more additions of Neu5Ac. Owing to the need for high amounts of nucleotide sugars and glycosylation potential in HMO biosynthesis, a suitable host must have robust sugar metabolism capable of managing the metabolic burden of HMO production.

Unlike many microbes used in commercial fermentation, plants have evolved to create a wide range of glycans which encompass a diversity of nucleotide sugars from photosynthetically fixed CO_2_. As masters of sugar anabolism, plants are able to create vast amounts of complex oligosaccharides and polysaccharides^17^. This has led to commercial operations for the production of prebiotic oligosaccharides from plant biomass, such as ß-glucan, xylooligosaccharides, inulin or soy oligosaccharides^18–21^. Many of these products can either be purified or directly consumed as a food, providing an easy means of ingestion. Additionally, plants can be grown in open fields, requiring minimal inputs, limiting the need for expensive substrates and axenic conditions^22^. The robust sugar metabolism of plants and ability to be grown at agricultural scales make plants an ideal platform for the large-scale production of HMOs.

Owing to the unique advantages of plants as a platform for carbohydrate production, we tested their ability to produce a range of HMOs using both transient and stable expression in *Nicotiana benthamiana*. Here, we report in planta production of neutral, fucosylated and acidic HMOs showcasing the intrinsic advantages of a plant-based production platform. Furthermore, we show that plant-produced HMOs provide selective growth of key bifidobacteria, indicating their potential prebiotic efficacy. Finally, we assess the economic viability of HMO production in planta compared to current microbial platforms.

## Results

### Production of all three HMO classes in planta

Production of HMOs requires the expression of glycosyltransferases capable of creating specific glycosidic linkages. While HMO biosynthesis in humans takes place in the Golgi by means of a largely unknown pathway^23^, various microbial enzymes are capable of generating HMOs in the cytosol. To produce HMOs in plants, we localized bacterial HMO biosynthetic enzymes to the cytosol for the production of neutral, fucosylated and acidic HMOs (Fig. 1c). We tested these pathways through transient expression in *N. benthamiana*. Transient expression permits relatively high throughput screening of biosynthetic pathways in planta by injecting strains of *Agrobacterium tumefaciens* into plant leaves, allowing the *Agrobacterium* to insert genes for HMO biosynthetic genes into the plant cell^24^. Leaves transiently expressing HMO biosynthetic pathways were subjected to liquid–liquid extraction, C18 solid-phase extraction (SPE) and porous graphitic carbon (PGC) SPE before characterization by mass spectrometry (MS) (Supplementary Fig. 1).

Neutral HMOs function as the core scaffolds of other more complex HMOs (fucosylated and acidic); thus, we first targeted the type I and type II neutral HMO core structures, lacto-*N*-tetraose (LNT) and lacto-*N*-neotetraose (LNnT). Expression of a neutral HMO biosynthetic pathway using two ß-1,4-galactosyltransferases (*GalTPM1141* (ref. ^25^), *Hp0826* (ref. ^26^)), one ß-1,3-galactosyltransferase (*Cvß3GalT*^27^) and one ß-1,3-*N*-acetylglucosaminyltransferease (*NmLgtA*^28^) resulted in the production of lactose and various neutral HMOs with degrees of polymerization ranging from three to seven. Notably, *N. benthamiana* transiently expressing this pathway produced the tetrasaccharides LNT (*m*/*z* 708.2559*)* and LNnT (*m*/*z* 708.2559), which represent principal type I and type II HMOs in human milk, respectively^29^ (Fig. 1d and Supplementary Table 1). Additionally, we identified the production of larger neutral oligosaccharides with varying degrees of polymerization using tandem mass spectrometry (MS/MS) fragmentation to determine the number of hexose and HexNAc sugars (Supplementary Table 1). This included several neutral isomers of pentasaccharides and heptasaccharides (Supplementary Table 1). Our findings demonstrate that plants have the ability to produce previously inaccessible oligosaccharides with various degrees of polymerization which may expand HMO functional bioactivity.

Following the success of generating type I and type II neutral HMOs, we examined the ability of plants to decorate neutral HMOs with fucose, as fucosylated HMOs are the most abundant class of HMO in human milk^15^. We transiently expressed an α-1,2-fucosyltransferase (*Te2FT*^30^) alongside the neutral HMO biosynthetic pathway to produce the most abundant fucosylated HMOs in human milk: 2′-fucosyllactose (2′FL) (*m*/*z* 489.1819) and lacto-*N*-fucopentaose I (LNFPI) (*m*/*z* 854.3136) (Fig. 1d and Supplementary Table 1). Additionally, several fucosylated hexasaccharide isomers were identified by *m*/*z* and MS/MS fragmentation (Supplementary Table 1). While the structure of each isomer could not be determined, each is composed of four hexoses, one HexNAc and one deoxyhexose, indicating that either LNFPI can be further decorated with hexose sugars or pentasaccharide neutral HMOs can be decorated with additional fucose.

While neutral and fucosylated HMOs represent most HMOs in human milk, acidic HMOs constitute the last main class found in mammalian milks which provide unique bioactivities as a result of the presence of *N*-acetylneuraminic acid^31^. Plants do not natively produce the donor molecule for production of acidic HMOs, CMP-Neu5Ac. To produce acidic HMOs, we simultaneously expressed the neutral HMO biosynthetic pathway, sialyltransferases and a mammalian pathway for the production of CMP-Neu5Ac (Supplementary Fig. 2)^32^_._ Expression of an α-2,6-sialyltransferase (*St6*; ref. ^33^) alongside the neutral HMO biosynthetic pathway produced the acidic trisaccharide, 6′-sialyllactose (6′SL) (*m*/*z* 634.2191), of type II acidic HMO, sialyllacto-*N*-neotetraose c (LSTc) (*m*/*z* 999.3505) (Fig. 1d and Supplementary Table 1). Expression of an α-2,3-sialyltransferase (*PmST3*; ref. ^34^) with the neutral HMO biosynthetic pathway produced a myriad of acidic HMOs, such as the acidic trisaccharide, 3′-sialyllactose (3′SL) (*m*/*z* 634.2187) and the acidic pentasaccharide, LSTd (*m*/*z* 999.3510) (Fig. 1d and Supplementary Table. 1). In addition to making several LST isomers in vivo, six isomers of acidic hexasaccharides were identified using *m*/*z* and MS/MS fragmentation. Each isomer was composed of four hexoses, one HexNAc and one Neu5Ac (Supplementary Table 1). Together, these results show the ability of plants to produce all three classes of HMOs combinatorially or simultaneously (Supplementary Fig. 3), including type I and type II structures, marking the greatest diversity of HMOs made in a single heterologous organism.

### Optimized production of complex fucosylated HMOs

**Fig. 2:**
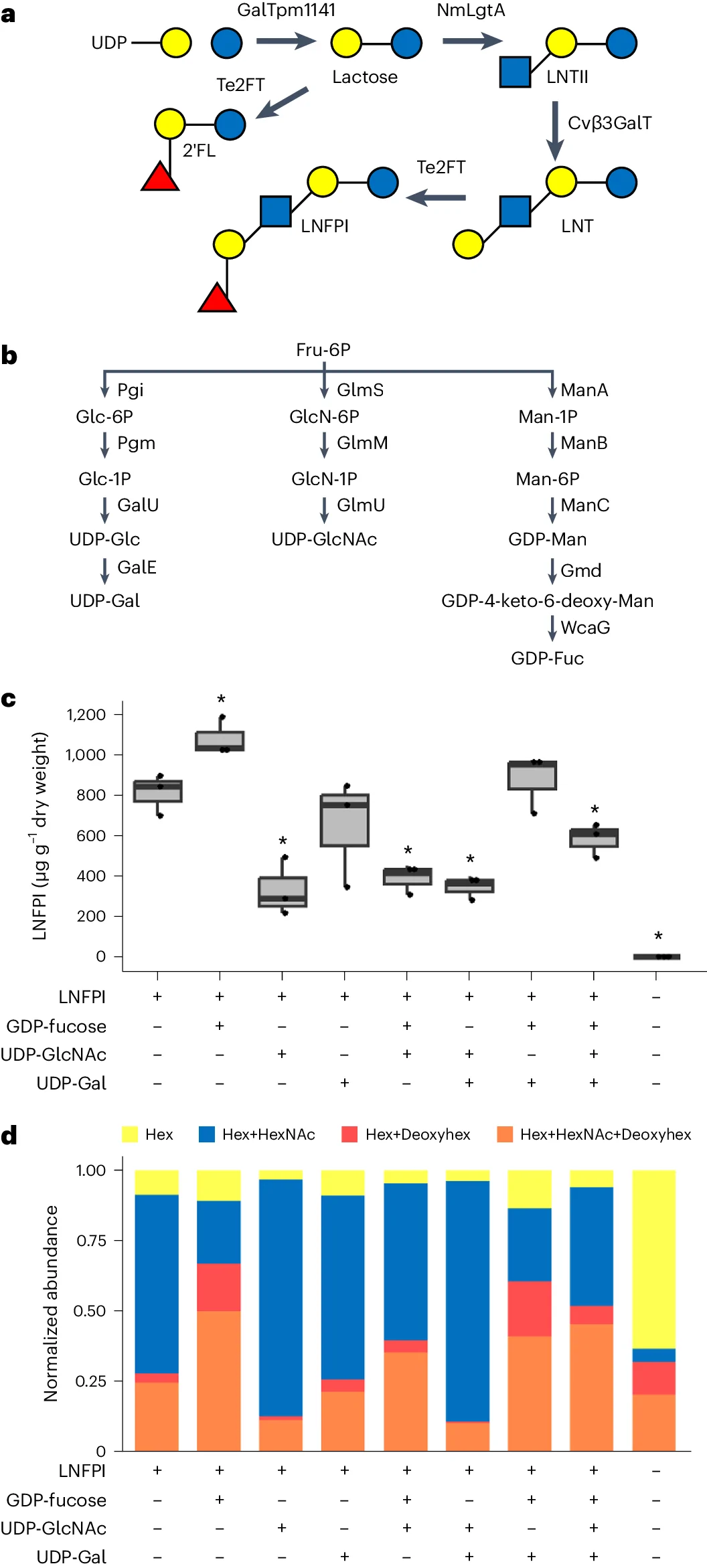
Manipulation of nucleotide sugar biosynthetic pathways modulates HMO profiles in planta. **a**, HMO biosynthetic pathway expressed for the production of LNFPI. **b**, Nucleotide sugar biosynthetic pathways expressed with LNFPI pathway. **c**, Quantification of LNFPI production through expression of LNFPI biosynthetic pathway alongside combinatorially expressed nucleotide sugar biosynthetic pathways using an internal calibration curve obtained with an Agilent 6530 Accurate-Mass Q-ToF MS. The middle bar represents the median. Upper and lower whiskers correspond to the largest and smallest values within 1.5 × the interquartile range, respectively. Upper and lower hinges represent the third and first quartiles, respectively. Statistical analysis was conducted using a heteroscedastic two-tailed Student’s *t*-test with the LNFPI pathway expressed alone used as the reference group. *P < 0.05. *P* values are: LNFPI + fucose, 0.030; LNFPI + GlcNAc, 0.012; LNFPI + fucose + GlcNAc, 0.01; LNFPI + GlcNAc + Gal, 0.01; LNFPI + fucose + GlcNAc + Gal, 0.043. A sample size of three leaves was used for each experiment. **d**, Effect of nucleotide sugar biosynthetic pathway overexpression on HMO profile produced using the LNFPI pathway. Values reflect normalized peak area. Hexose, HexNAc, deoxyhexose (Deoxyhex) determined using *m*/*z* and MS/MS fragmentation. We performed mass spectral analysis on an Agilent Q-ToF MS.

Microbial production platforms suffer from an inability to produce HMOs with higher degrees of polymerization at large scales, leaving many larger, more complex HMOs understudied. LNFPI is a fucosylated pentasaccharide that is the second most abundant fucosylated HMO. Despite its high abundance in breast milk, LNFPI has remained recalcitrant to fermentative production in microbes, limiting efforts to study its potential health benefits. Therefore, we sought to optimize the production of LNFPI in planta by overexpressing the requisite nucleotide sugar biosynthetic pathways. We transiently expressed the biosynthetic pathway for LNFPI (Fig. 2a) alongside pathways for the production of UDP-galactose, UDP-*N*-acetylglucosamine (UDP-GlcNAc) and GDP-fucose and quantified LNFPI production (Fig. 2b). Expression of the LNFPI pathway with the GDP-fucose pathway increased production of LNFPI by 32.9% (1,075.03 μg g^−1^ of dry weight) compared to the expression of the LNFPI pathway alone (808.91 μg g^−1^ of dry weight), indicating that GDP-fucose is limiting in *N. benthamiana* (Fig. 2c). Surprisingly, overexpression of the GDP-fucose pathway also resulted in the production of lactodifucotetraose (LDFT) (*m*/*z* 635.2394) and lacto-*N*-difuco-hexaose I (LNDFHI) (*m*/*z* 1,000.3720) (Supplementary Table 1) despite not expressing an α-1,3- or α-1,4-fucosyltransferase, indicating the presence of native plant fucosyltransferases capable of glycosylating HMOs. Overexpression of all other nucleotide sugar pathway combinations resulted in similar or lower levels of LNFPI production compared to expression of the LNFPI pathway alone.

The overall profile of oligosaccharides produced was altered by overexpressing nucleotide sugar biosynthetic pathways (Fig. 2d). The number of hexose (Glc, Gal), HexNAc (GlcNAc) and deoxyhexose (Fuc) sugars in each oligosaccharide identified was determined through identification by means of *m*/*z* and MS/MS fragmentation and peak area was normalized using a LNFPI calibration curve. Overexpression of the UDP-GlcNAc and LNFPI pathways increased the relative amount of neutral oligosaccharides containing a hexose and HexNAc compared to expression of the LNFPI pathway alone (Fig. 2d). Overexpression of the GDP-fucose and LNFPI pathways resulted in a shift in the overall oligosaccharide composition, favouring the production of oligosaccharides containing at least one deoxyhexose, indicating an increase in the level of fucosylated oligosaccharides (Fig. 2d). These results demonstrate that tailoring the availability of nucleotide sugars enables control over the ratio of HMOs produced.

**Fig. 3:**
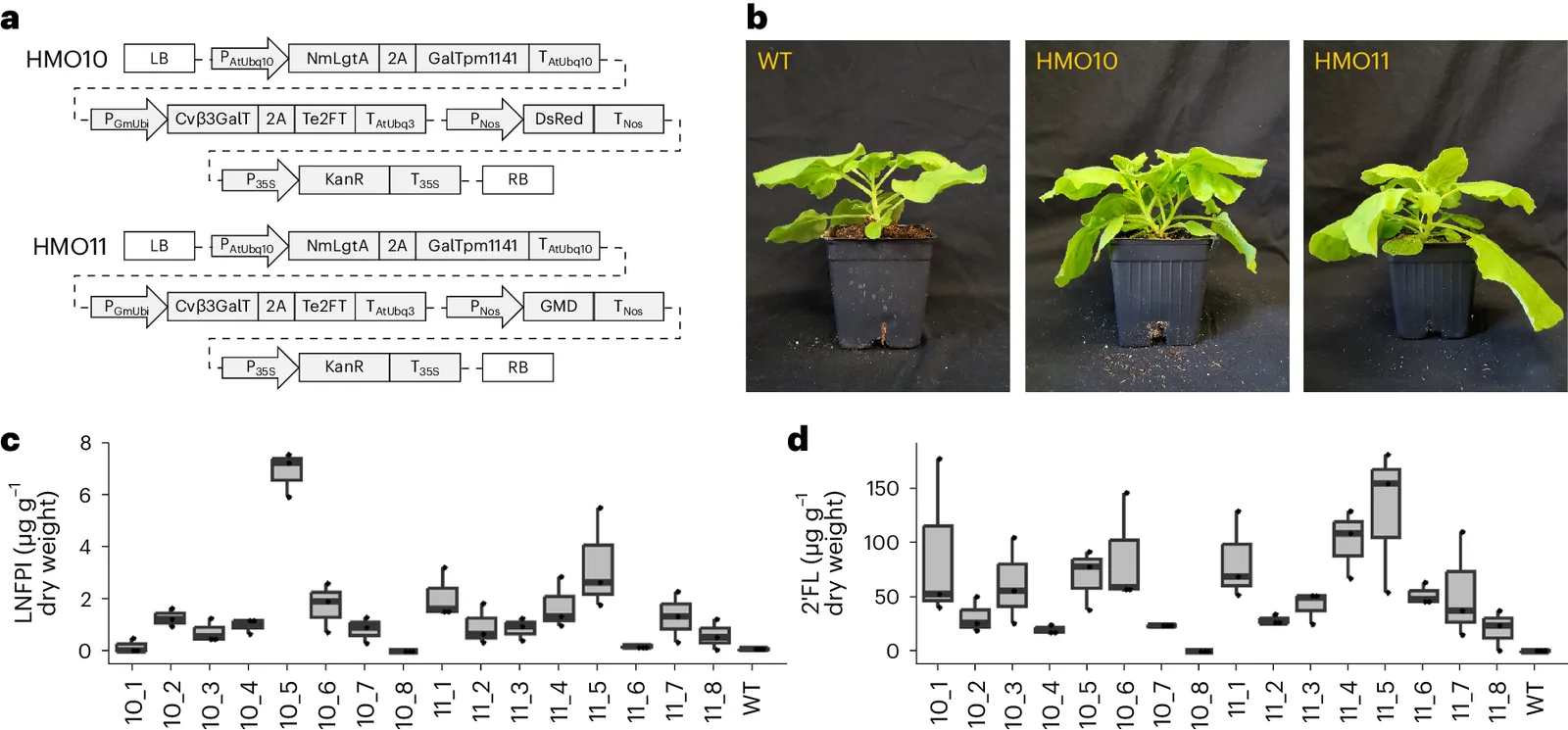
Production of HMOs in stably transformed plants. **a**, Constructs used in creation of stable lines containing biosynthetic enzymes for the production of LNFPI. **b**, Photos of 4-week-old transgenic *N. benthamiana*. **c**, Concentration of LNFPI produced in leaves of each stable line. **d**, Concentration of 2′FL produced in leaves of each stable line. For quantification, three leaves from each plant were analysed separately. Quantification of LNFPI and 2′FL obtained with a Thermo Fisher Scientific Q Exactive Mass spectrometer. LB, left border; 2A , 2A peptide; RB, right border; P_*x*_, promoter; T_*x*_, terminator; WT, wild type. The middle bar represents the median. Upper and lower whiskers correspond to the largest and smallest values within 1.5 × the interquartile range, respectively. Upper and lower hinges represent the third and first quartiles, respectively. A sample size of three leaves was used for each experiment.

Because scaling HMO production in plants requires the growth of stably transformed plants, we developed transgenic lines of *N. benthamiana* expressing the LNFPI biosynthetic pathway. We generated two constructs for the constitutive production of 2′FL and LNFPI in transgenic *N. benthamiana* (Fig. 3a). HMO10 contains genes that encode four enzymes required to produce lactose, 2′FL, LNTII, LNT and LNFPI connected by means of 2A peptides^35^ to allow several coding sequences to be driven by a single constitutive promoter. To explore the effects of overexpressing portions of the GDP-fucose pathway, we also generated stable lines expressing HMO11, which contains a GDP-d-mannose-4,6-dehydratase (Gmd^25^) from the GDP-fucose pathway. Gmd transiently expressed alongside the neutral HMO pathway altered the HMO profile of plants in a similar way to expression of the full GDP-fucose pathway (Supplementary Fig. 4).

Transgenic T0 HMO-producing stable lines were assessed for fucosylated HMO yield. Most transgenic plants showed no drastic phenotypes compared to wild-type plants (Fig. 3b) and we conducted quantitative PCR with reverse transcription (RT–qPCR) analysis to confirm the expression transgenes (Supplementary Fig. 5). LNFPI and 2′FL were detected in leaves of transgenic *N. benthamiana* expressing both HMO10 and HMO11. Highest producing LNFPI lines accumulated an average concentration of 6.88 µg g^−1^ dry weight (Fig. 3c). Leaves from HMO11 no. 5 produced the highest concentration of 2′FL, reaching an average concentration of 130.35 µg g^−1^ dry weight (Fig. 3d). The low abundance of LNFPI in stable lines compared to transient expression could indicate that the ß-1,3-*N*-acetylglucosaminyltransferase and ß-1,3-galactosyltransferase suffer from altered activity due to the presence of 2A peptides or lower expression. Together, these results show the ability to produce and optimize a diversity of HMOs from photosynthetically fixed CO_2_, laying the foundation for future efforts to create high-HMO-yielding transgenic plants for commercial HMO production.

### Purification and functional characterization of HMOs from plants

Mixtures of prebiotic sugars can have varying effects on the enrichment of beneficial gut microbes^36^. Therefore, we sought to assess the bifidogenic activity of extracts from HMO-producing plants; however, crude plant extracts can contain chemicals that interfere with bacterial growth assays, such as simple sugars (glucose, fructose and sucrose) and antimicrobial specialized metabolites. Therefore, we developed a method to extract and purify HMOs from *N. benthamiana* transiently expressing the biosynthetic pathways for LNFPI (Fig. 2a) and GDP-fucose (Fig. 2b) using an optimized extraction and purification process. Briefly, we performed a water extraction, yeast fermentation to remove simple sugars and a two-step resin adsorption with polyvinylpolypyrrolidone (PVPP) and C18 SPE. This resulted in an HMO-rich extract that contained negligible amounts of simple sugars and phenolic compounds (Supplementary Table 3). The HMO extract contained target fucosylated HMOs (Supplementary Fig. 6), including LNFPI, 2′FL and LNDFHI (Supplementary Table 4). The extract also contained a variety of additional oligosaccharides without assigned structures, which were composed of combinations of hexose, deoxyhexose and HexNAc sugars (Supplementary Fig. 6). These represent potentially non-natural oligosaccharide structures which could provide potential health benefits. Overall, these results show the ability to isolate HMOs from plants, improving their promise as an HMO production platform.

**Fig. 4:**
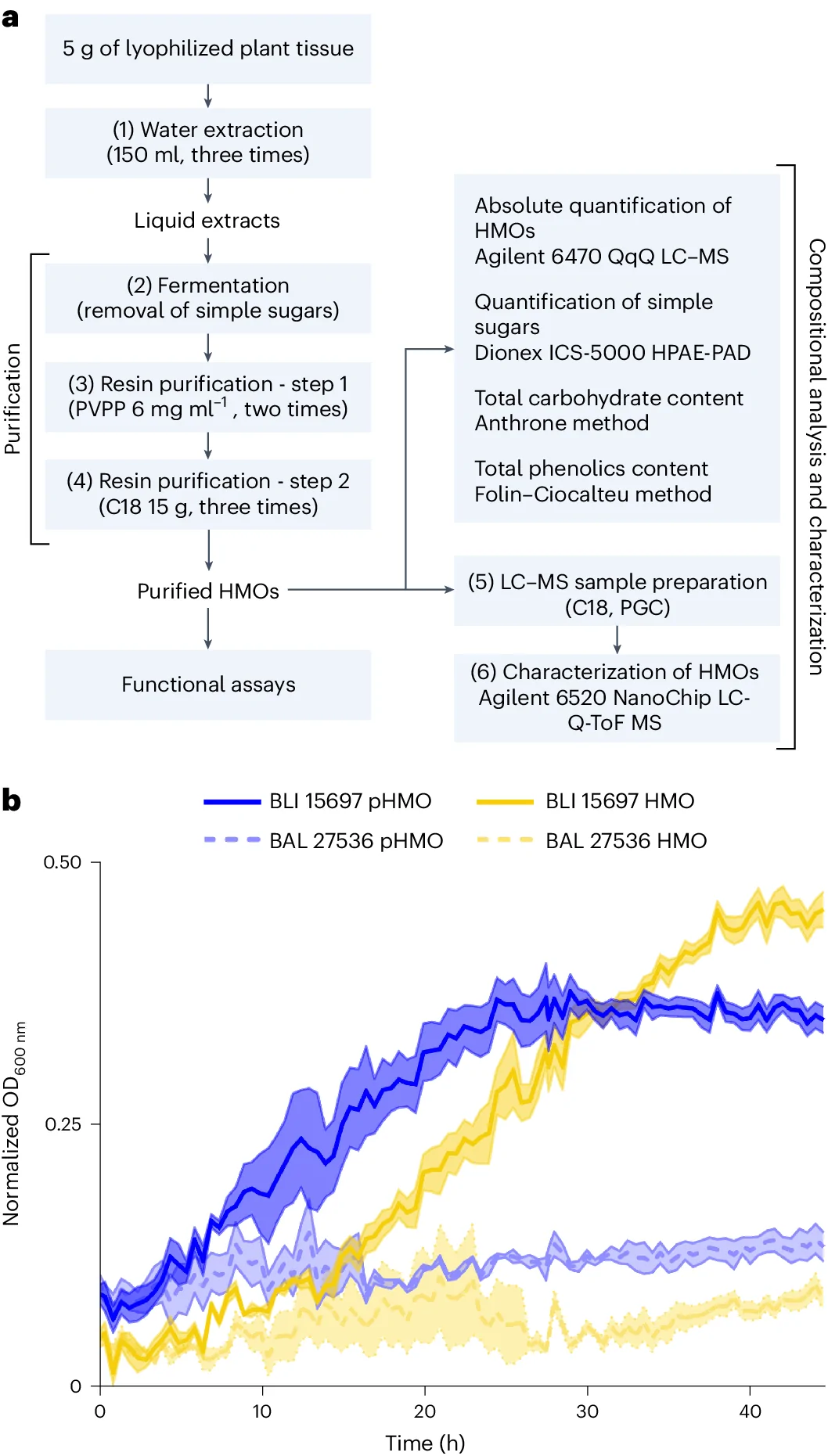
Optimized purification protocol developed for functional analysis of plant-produced HMOs. **a**, Workflow of extraction, purification and characterization of HMOs from *N. benthamiana* leaves. **b**, Growth curves of HMO-consuming (BLI 15697) and control (BAL 27536) strains in media supplemented with HMO isolated from breast milk (HMO) or HMOs isolated from plants (pHMO). Error bars represent standard deviation.

To assess the bifidogenic activity of plant-produced HMOs, we conducted growth assays to compare the effects of plant-produced HMOs to HMOs derived from human milk. We chose to assess the effects of plant-derived HMOs on *Bifidobacterium longum* subsp. *infantis* ATCC 15697 (BLI 15697) as it is a known HMO consumer^37^. We also included *Bifidobacterium animalis* subsp. *lactis* ATCC 27536 (BLAC 27536) as a negative control which does not consume HMOs^38^ but will grow on simple sugars that could be present in plant extracts or human milk. BLI 15697 grown in media containing plant-derived HMOs showed increases in optical density OD_600 nm_ similar to that of BLI 15697 in HMO isolated from human milk, demonstrating that plant-produced HMOs possess the same selective bifidogenic activity as HMOs isolated from human milk (Fig. 4b). As expected, BLAC 27536 showed no growth in either plant-derived HMOs or HMO isolated from human milk, indicating that both extracts contained a minimal amount of simple sugars present (Fig. 4b). Together, these results demonstrate the ability of purified, plant-produced HMOs to mimic the bifidogenic activity of HMOs produced in humans.

#### Economic viability of plant-based HMO production

Plant-based HMO production can be commercially viable if it demonstrates cost-competitive or cost-advantage with current state-of-the-art production routes. To assess the economic viability of HMO production in a commercially relevant crop, we developed process models and compared the cost of HMO production in plants and microbes. To do this, we performed technoeconomic analysis (TEA) of the theoretical production of LNFPI. In the plant system, we adopted typical cellulosic biorefinery design using biomass from sorghum to coproduce HMOs along with biofuel because coproducing value-added bioproducts in biorefineries is a promising approach to maximize the use of biomass and hence improve the economics of biorefineries^39,40^. We assume that biomass sorghum can accumulate 0.31% dry weight of LNFPI in the entire biomass, as this was our highest yield following purification of LNFPI (Supplementary Table 4). We also developed process models and conducted TEA for the HMOs in *Escherichia coli* using the established processes and the highest reported yields of LNFPI^41^ from peer-reviewed papers at the time of conducting this TEA. The comparison between plant and microbial systems to produce the same product aims to provide indepth understanding of the cost–benefits of the systems.

**Fig. 5:**
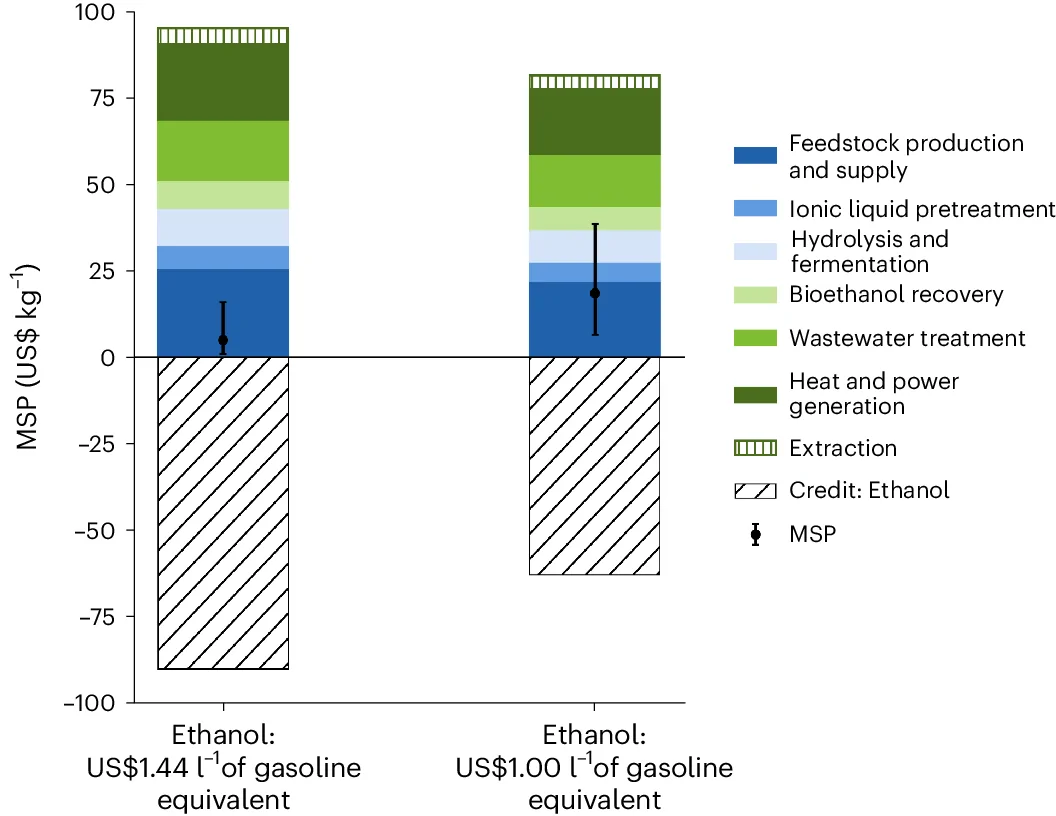
Plant-based platform improves the economics of producing the HMO, LNFPI. Estimated MSP of LNFPI produced using biomass sorghum as a model production platform in two bioethanol price scenarios. Error bands represent final values calculated with ±20% of input parameters.

TEA results indicate that producing LNFPI from the plant system is economically favourable compared to the microbial system (Supplementary Fig. 7). In the plant system, the minimum selling prices (MSPs) of LNFPI are US$4.9 kg^−1^ when selling ethanol at the cellulosic ethanol selling price and US$18.4 kg^−1^ when ethanol is sold at the target fuel price, respectively (Fig. 5). However, microbial-based LNFPI results in MSP of US$45.0 kg^−1^ with downstream recovery and purification being the largest cost contributor, followed by the cost of glucose (Supplementary Fig. 7b). The high cost obtained in the microbial system is largely due to its extremely low highest reported yields (0.48%) and recovery rate after bioconversion (62%). These results indicate that when microbial hosts are unable to produce HMOs at a comparable yield, plants may be a cost-effective bioplatform for high-value products. In addition to relative cost advantages of plant systems, using biomass as the feedstock to coproduce high-value compounds and biofuel offers considerable environmental benefits because biomass can absorb CO_2_ from the atmosphere during its growth^42^. Although current HMO yields in stable lines of the model plant *N. benthamiana* (Fig. 3) are below the yields in transiently expressing tissue, high yields could be achieved by optimizing the HMO constructs used for the production of stable lines, finding an optimal crop species for HMO production and optimizing growing conditions.

## Discussion

Human milk oligosaccharides are major contributors to the bioactive properties of human milk^1,2^. While the unique bioactivities of few HMOs have been investigated^3,8–11^, over 100 HMOs remain to be studied owing to lack of access to material, representing a wealth of potential bioactive molecules. Here, we report the production of all three classes of HMOs in planta. Notably, expression of HMO biosynthetic pathways in planta created a variety of complex HMOs, including oligosaccharides that at present are not produced in microbial platforms, which indicates plants could serve as a platform for the production of a range of HMOs which are not currently producible in microbial hosts. Furthermore, we optimized the production of both specific HMOs and HMO classes by overexpressing nucleotide sugar biosynthetic pathways. The diversity of HMOs produced from inserting a relatively small number of genes shows the ability of plants to generate complex sugars. Transient expression serves as a viable platform for testing HMO biosynthetic genes and small-scale production of HMOs for functional validation. Optimized l, laboratory-scale purification methods enabled the isolation of oligosaccharides, including HMOs, from plant tissue, showing the potential of plants as an industrial source of HMOs. HMOs purified from plants provided selective bifidogenic activity, indicating that they would serve as a potent prebiotic in vivo. Future pathway engineering could yield biosynthetic pathways for the production of all HMOs present in human milk, including complex, branched HMOs. This would enable the study and potential consumption of bioactive HMOs that are currently unavailable.

Despite the field of plant synthetic biology being in its nascent stages, plants have the potential to produce compounds of interest at lower costs than microbial platforms because of their intrinsic and unique metabolic capabilities^39^. Additionally, plants are capable of using atmospheric carbon dioxide during their growth cycle to produce target compounds and biofuels, improving the sustainability of target compound production. We demonstrate the ability of stably transformed plants to produce two HMOs that are abundant in breast milk of most mothers, 2′FL and LNFPI. While the feasibility of purifying HMOs from plants at industrial scales still needs to be validated, our TEA results indicate that HMO production in commercially relevant crops has the potential to be a more cost-effective platform than microbial production for complex HMOs.

As the demand for HMOs increases because of the growing infant formula and adult prebiotic markets, plants may emerge as a cost-competitive and sustainable platform for the production of diverse HMOs at agricultural scales. Furthermore, the diversity of plant-produced HMOs will provide researchers with access to HMOs that were previously inaccessible. Since the structure of an HMO determines its bioactivity, this could lead to the discovery of HMOs that treat various gastrointestinal illnesses. Additionally, production of HMOs in plants could permit direct consumption as food by directly ingesting the plant or products made from the plant. Such a product may serve as a consumable source of prebiotic HMOs for humans or be added to forage crops for animal consumption. Overall, the production of HMOs in planta provides the opportunity to simultaneously improve the scale of HMO production and expand the diversity of HMOs available to improve the gastrointestinal health of infants and adults.

## Methods

### Plant growth

*N. benthamiana* was grown from seed in 3.5 inch square pots in a controlled environment facility. Plants were grown with a 12 h/12 h day/night cycle at ~700 µmol of photons per m^2^ s^−1^. Daytime growth chamber temperatures were kept at 26 °C. Night-time growth chamber temperatures were kept at 25 °C. Relative humidity in the growth chamber was kept between 60% and 75%.

### Plasmid construction and transient expression

For transient expression, the native sequences for candidate glycosyltransferases were PCR amplified and cloned into the binary vector, PMS057 (ref. ^43^), using Golden Gate assembly^44^, Gibson assembly^45^ or restriction-ligation (see Supplementary Table 5 for sequences). XL1-blue *E. coli* cells were transformed with the assembled plasmids via heat shock^46^. Transformed cells were selected by plating cells on Lysogeny broth (LB) agar plates containing 50 µg ml^−1^ of kanamycin. Plasmid assembly was confirmed by means of miniprep and Sanger sequencing (Azenta). *A. tumefaciens* str. GV3101 was transformed using sequence-verified plasmids by electroporation^47^. Transformed colonies were selected using LB agar plates containing 50 µg ml^−1^ of kanamycin, 50 µg ml^−1^ of rifampicin and 10 µg ml^−1^ of gentamicin. *A. tumefaciens* str. GV3101 harbouring individual candidate glycosyltransferases were grown in LB overnight to OD_600 nm_ (VWR, V-1200) of 0.8–1.2. The cultures were centrifuged at 4,000*g* for 10 min and the supernatant was decanted. Cell pellets were resuspended in infiltration media (10 mM MES, 10 mM MgCl_2_, 500 µM acetosyringone, pH 5.6) and incubated at room temperature for 1 h with gentle rocking (Thermolyne, VariMix). *A. tumefaciens* strains harbouring each glycosyltransferase were mixed in equal amounts alongside a strain harbouring the p19 silencing suppressor^48^ to reach a final OD_600 nm_ of 0.5. *A. tumefaciens* mixtures were injected into the abaxial side of a leaf on a 4-week-old *N. benthamiana* using a needleless syringe. Each experiment was performed with three biological replicates.

For the production of stable lines, HMO10 and HMO11 constructs were generated through a multipart Golden Gate assembly containing subcloned transcriptional units. Assembled plasmids were transformed and sequence verified as described above. *N. benthamiana* was transformed using *A. tumefaciens* str. EHA105 harbouring HMO10 or HMO11 by the UC Davis Plant Transformation Facility.

#### Quantitative PCR with reverse transcription

Total messenger RNA was extracted using E.Z.N.A. plant RNA kit (Omega Bio-tek) following manufacturer’s directions using the RB lysis buffer variation and on-column DNase digestion; complementary DNA synthesis was achieved with SSIV Vilo IV kit using random hexamers (Thermo Fisher Scientific). Quantitative PCR was performed using a CFX96 Real-Time thermocycler (Bio-Rad) programmed for detection of SYBR intercalating dye with the following temperature programming: 95 °C for 3 min, then 95 °C for 30 s, 60 °C for 45 s, repeated 34 times, then a gradual increase from 65 °C to 95 °C at 0.5 °C per minute to generate melt curves. Sso-Advanced Universal SYBR Green Supermix (Bio-Rad) was used for qPCR amplification. A previously validated primer set was used to amplify EF1α for internal normalization, primers for target genes were designed with Benchling’s qPCR primer design wizard and synthesized by IDT. One target gene from each transcriptional unit was chosen for both constructs (Supplementary Table 6). Melt curves for the product of all primer sets were unimodal and steep, suggesting only a single product was formed for each primer set. No reverse-transcriptase controls showed no amplification within the dynamic range of samples, confirming the efficacy of DNAse treatment and no template controls instituted at the beginning of RNA extraction with no plant matter and kept in parallel with real samples throughout all molecular steps did not amplify, confirming lack of contamination with extraneous DNA. Normalized relative expression was calculated using the ∆∆Cq method and normalized by setting the average level of amplification in the wild-type samples as 1.

### HMO extraction for identification of HMOs from individual leaves

*N. benthamiana* leaves transiently expressing HMO biosynthetic enzymes were harvested 5 days after infiltration. Three leaves of *N. benthamiana* stable lines transformed with HMO10 and HMO11 were harvested at 4 weeks old. Following harvest, vasculature was removed and leaves were frozen in liquid nitrogen and lyophilized (Labconco, Freezone 4.5) for 2 days. Lyophilized leaves were homogenized via a bead mill (Retsch, MM400) at 20 Hz for 10 min. Oligosaccharides were extracted from 20 mg of lyophilized leaf tissue by ethanol precipitation. To each sample, 1 ml of 80% ethanol was added before homogenization on a bead mill at 10 Hz for 1 min. Samples were then precipitated overnight at −20 °C and centrifuged at 10,000*g* for 15 min. The supernatant was transferred to a 2 ml screw-cap tube. The pellet was washed twice by adding 500 μl of 80% ethanol, homogenizing via bead mill for 1 min and centrifuging at 10,000*g* for 15 min. The supernatant and washes were combined and dried in a vacuum centrifuge (Genevac EZ-2, SP Scientific). Dried supernatants were reconstituted in 200 μl of water and subjected to both C18 and PGC SPE (Thermo Fisher Scientific) in 96-well plate format. C18 cartridges containing 25 mg of stationary phase were first conditioned by two additions of 250 μl of acetonitrile (ACN) followed by four additions of 250 μl of water. Samples were then loaded and eluted with two volumes of 200 μl of water. PGC cartridges containing 40 mg of stationary phase were conditioned by addition of 400 μl of water, 400 μl of 80% (v/v) ACN and water, followed by two volumes of 400 μl of water. The sample eluate from C18 SPE was then loaded, washed thrice with 500 μl of water and eluted using two volumes of 200 μl of 40% (v/v) ACN and water. The purified extracts were dried in a vacuum centrifuge and reconstituted in 100 μl of water before injecting 5 μl for liquid chromatography mass spectrometry (LC–MS) analysis.

For quantification of LNFPI in Figs. 2 and 3, LNFPI at known concentrations was added to the extraction solution. The extraction solution was then used on wild-type *N. benthamiana*. This was done to ensure accuracy of HMO quantification by accounting for HMO losses in the extraction processes and ion suppression that could occur due to the plant metabolites present.

### LC–MS analysis of HMOs from individual leaves

For initial screening, chromatographic separation was carried out using a Thermo Scientific Vanquish UHPLC system equipped with a Waters BEH C18 Amide column (HILIC) (1.7 µm, 100 mm × 2.1 mm). A 10 min binary gradient was used based on ref. ^49^: 0.0–4.0 min, 25–35% A; 4.0–8.50 min, 35–65% A; 8.50–8.70 min, 25% A. Mobile phase A consisted of 3% ACN (v/v) in water with 0.1% formic acid and mobile phase B consisted of 95% ACN (v/v) in water with 0.1% formic acid.

For identification of HMOs produced, we performed LC–MS analysis using a Thermo Scientific Vanquish 3000 UPLC system connected to Thermo Scientific Q Exactive mass spectrometer. Chromatographic separation was carried out using a Hypercarb PGC column (5 µm, 150 mm × 1 mm, Thermo Scientific). A 40 min binary gradient using 3% ACN in water containing 0.1% formic acid (Solvent A) and 90% (v/v) ACN in water containing 0.1% formic acid was performed as follows: 100% A, 0–2.5 min; 100–84% A, 2.5–15 min; 84–42% A, 15–20 min; 42–0% A, 20–22 min; 0% A, 22–28 min; 0–100% A, 28–30 min; 100% A, 30–40 min.

For identification of HMOs, the Q Exactive mass spectrometer equipped with an electrospray ionization source was operated in positive ionization mode with the following parameters: scan range *m*/*z* 133.4–2,000; spray voltage 2.5 kV, capillary temperature 320 °C, aux gas heater temperature 325 °C, sheath gas flow rate 25, aux gas flow rate 8, sweep gas flow rate 3. MS/MS analysis was performed using stepped collision energies of 20, 30, 40 eV. MsDIAL was used for data analysis^50^.

For quantification of LNFPI and HMO profiling, mass spectral analysis was carried out on an Agilent 6530 Accurate-Mass Q-ToF MS operated in positive mode using data-dependent acquisition. The gas temperatures were held at 150 °C. The fragmentor, skimmer, octopole and capillary were operated at 70, 55, 750 and 1,800 V, respectively. The collision energy was based on the empirically derived linear formula (1.8 × (*m*/*z*/100) − 3.6). The reference mass used for calibration was *m*/*z* 922.009798. The Agilent MassHunter Qualitative software was used for data analysis. Oligosaccharides were identified using an inhouse library, their MS/MS spectra and comparison to either authenticated standards or a pool of HMOs of known composition.

### Extraction and purification of HMOs from pooled leaves

Five grams of lyophilized and ground *N. benthamiana* leaves transiently expressing the LNFPI and GDP-fucose biosynthetic pathways was mixed with 150 ml of water and agitated for 15 min at room temperature in a stirring plate. The mixture was centrifuged at 4,000*g* for 5 min and the supernatant was separated. The extraction was repeated two more times, combining the supernatant each time. The final supernatant was filtered using a 0.22 µm Millipore Steritop vacuum filter. The extraction process was carried out in duplicate to ensure reproducibility.

Yeast fermentation was carried out to eliminate simple sugars (glucose, sucrose and fructose) from the extracts^51^. Briefly, autoclaved extracts were inoculated with 0.4 g l^−1^ of commercial active dry yeast *Saccharomyces cerevisiae*^52^ (UCD 522 Montrachet, Lallemand) at 30 °C, 150 rpm for 24 h (Excella E24 Incubator Shaker Series, New Brunswick Scientific). After 24 h, the samples were centrifuged at 4,000*g* for 5 min and filtered using a 0.22 µm Millipore Steritop vacuum filter to remove the yeast. Samples were concentrated using a vacuum concentrator (Genevac miVac Centrifugal Concentrator) at room temperature and frozen until their purification.

PVPP (Sigma-Aldrich) was used to bind phenolic compounds within the sample following a previous protocol^52^. Briefly, 3 g of PVPP was conditioned by mixing it with 100 ml of 12 M HCl at 100 °C for 30 min with constant stirring in a stirring plate. After cooling off, the slurry was centrifuged at 4,000*g* for 5 min and filtered using a 0.22 µm Millipore Steritop vacuum filter. Subsequently, the PVPP was washed with nanopure water until the flow-through reached pH 7. Activated PVPP was mixed with water to a final concentration of 20 mg of PVPP per ml.

PVPP suspension was added to the extracts at a concentration of 6 mg of PVPP per ml and agitated at room temperature for 15 min on a stirring plate. After the time had elapsed, the sample containing the plant extracts and the PVPP was centrifuged at 4,000*g* for 5 min and filtered using a 0.22 µm Millipore Steritop vacuum filter to separate the PVPP containing the bound phenolics. To eliminate residual phenolics from the extracts, more PVPP was added to the supernatant (6 mg of PVPP per ml) and the process was repeated. The filtrate containing the oligosaccharides was concentrated and frozen until further purification.

Two SPE columns of 60 ml were packed with 15 g of Bondesil-C18, 40 µm suspended in 20 ml of ACN. After the ACN was drained, a frit was added to the C18. Before loading the samples, the columns were conditioned with three volumes of ACN and three volumes of nanopore water. PVPP-treated extracts were loaded onto the conditioned C18 columns and the oligosaccharides were washed with 250 ml of nanopure water divided into four washes. To ensure the complete removal of interfering compounds, C18 SPE was repeated two more times. The purified HMO fractions were dried in a vacuum concentrator (Genevac miVac Centrifugal Concentrator) at room temperature and frozen.

### Compositional analysis of plant material

Total carbohydrate content was assessed by the anthrone method with modifications^53^. In a 96-well microplate, 40 μl of purified and diluted extracts were combined with 100 μl of anthrone reagent (2 mg ml^−1^ (w/v) in cold 98% sulfuric acid) and mixed through pipette tip aspiration. The microplate was incubated for 3 min at 92 °C in a water bath followed by 5 min at a room temperature water bath and then 15 min in a 45 °C Thermolyne Benchtop muffle furnace (Thermo Fisher Scientific). The plate was cooled for 3 min at room temperature before measuring the absorbance with a SpectroMax M5 UV/Vis spectrophotometer (Molecular Devices) at 630 nm. Total carbohydrate quantification calculations were based on a glucose standard curve. Each plant extract was prepared in duplicate and each sample was further analysed in duplicate.

Total phenolic content of the extracts was determined according to the Folin–Ciocalteu spectrophotometric method as described by ref. ^54^.

Simple sugars (glucose, sucrose and fructose) were quantified by high-performance anion exchange chromatography with pulsed amperometric detection on a Thermo Fisher Dionex ICS-5000+ HPAE-PAD system based on a method described by ref. ^55^ with modifications. Diluted extracts (1:100, v/v or 1:1,000 in nanopure water) were filtered through a 0.2 mm syringe filter (Agilent Captiva Econo Filter, PES, 13 mm, 0.2 μm) into 2 ml vials with septa. The samples (25 μl) were injected into a CarboPac PA200 guard column (3 × 50 mm) and a CarboPac PA200 analytical column (3 × 250 mm) and chromatographic separation was carried out with a 12 min gradient elution (from 0.6% to 25% B in 12 min), 0.5 ml min^−1^ flow rate. The solvent system consisted of A: 100% water; and B: 200 mM sodium hydroxide. Calibration curves (correlation coefficient ≥0.999) were prepared using glucose, sucrose and fructose standards.

### Quantification of HMOs by QqQ LC–MS

Detection and quantitation of HMOs were performed using an Agilent 6470 triple quadrupole LC–MS system (QqQ LC–MS) equipped with an Advance Bio Glycan Map column (2.1 mm × 150 mm, 2.7 μm, Agilent). The mobile phase consisted of 10 mM ammonium acetate in 3% ACN, 97% water (v/v, pH 4.5; A) and 10 mM ammonium acetate in 95% ACN, 5% water (v/v, pH 4.5; B). The chromatographic separation was carried out at 35 °C with gradient elution at a flow rate of 0.3 ml min^−1^. The MS analysis was conducted in positive ion mode with source parameters as follows: the gas temperature was 150 °C at a flow rate of 10 l min^−1^; the nebulizer was 45 psi; the sheath gas temperature was 250 °C at a flow rate of 7 l min^−1^; capillary voltage was 2,200 V. See Supplementary Table 2 for gradient and multiple reaction monitoring transitions.

### Characterization of HMOs by LC-QToF-MS

Oligosaccharides were purified by a two-step SPE using C18 (HyperSep C18–96, 50 mg bed weight; Thermo Fisher Scientific) and PGC (HyperSep Hypercarb-96, 25 mg bed weight; Thermo Fisher Scientific)^56^. The samples were filtered (Captiva Premium Syringe Filter PES membrane, 4 mm diameter, 0.2 µm pore size, LC/MS certified) into 200 µl vials.

Individual oligosaccharide compositions were analysed with an Agilent 6520 NanoChip LC-QToF mass spectrometer. Oligosaccharides separation was achieved with a microfluidic high-performance liquid chromatography PGC chip containing an enrichment (4 mm, 40 nl) and an analytical (75 μl × 43 mm) column as well as a nanoelectrospray tip, using a binary solvent gradient of solvent A (5 mM ammonium acetate in 3% ACN, 97% water (v/v)) and solvent B (5 mM ammonium acetate in 90% ACN, 10% water (v/v)) based on a previously optimized method^55^. The gradient was 0–16% B at 0–20 min, 16–44% B at 20–30 min, 44–100% B at 30–35 min, 100% B at 35–45 min and 100–0% B from 45 to 45.1 min, followed by a 15 min re-equilibration of 100% A^57^. The mass spectrometer was operated in positive ionization mode with a range of *m*/*z* 320–2,500 and an electrospray capillary voltage of 1,800–1,900 V. Reference masses of *m*/*z* 922.009 and 1,221.991 provided continuous internal calibration. All samples were analysed using MS/MS with tandem fragmented peaks selected by the automated precursor selection of the six ions with highest signal intensity with a medium isolation width. The Q-ToF MS had a ramped collision energy slope of 0.02 based on *m*/*z* values with an offset of −3.5 V. The acquisition rate of 1 spectrum per s was used for both MS and MS/MS. Each spectrum was manually examined and molecular masses were confirmed with Agilent MassHunter Qualitative Analysis B.07.00 software using the molecular feature extraction and a maximum tolerance of 20 ppm.

### Bacterial strains and growth conditions

*B. longum* subsp. *infantis* ATCC 15697 and *B. animalis* subsp. *lactis* ATCC 27536 were cultured at 37 °C in a Coy vinyl anaerobic bubble with an atmosphere of 2.5% H_2_, ~5% CO_2_ and balance N_2_. Routine culturing was done with Difco MRS + 0.05% l-cysteine HCl (MRSC) and carbohydrate-specific culturing was done with modified MRS (mMRSC), which was prepared per litre as follows: 10 g of Bacto proteose peptone no. 3, 10 g of Bacto casitone, 5 g of Bacto yeast extract, 2 g of triammonium citrate, 5 g of sodium acetate trihydrate, 200 mg of magnesium sulfate hexahydrate, 34 mg of manganese sulfate monohydrate, 0.5 g of l-cysteine HCl and 1.063 g of Tween-80. Normally, 2 g of anhydrous dipotassium phosphate would also be added but it was precipitated by the plant HMO preparation.

### Growth curves

One colony was used to inoculate 1 ml mMRSC + dipotassium phosphate + 1% lactose monohydrate and incubated for 24 h. The growth curve inoculum was cultured by diluting the 24 h culture 1:100 in mMRSC + dipotassium phosphate + 1% lactose, then incubating for 12–15 h. The inoculum was prepared by washing the cells twice with one volume of 1× PBS. Growth curve cultures (160 μl) were contained in flat-bottomed, optically clear, 96-well, lidded plates and they had a final inoculum and sugar concentration of 1% in mMRSC. Lactose was the growth control substrate, water was the no-growth control substrate and pooled HMO^58^ was the HMO-growth control substrate. Cultures were done in triplicate. Wells were overlaid with 40 μl of sterile mineral oil and incubated in a BMG SpectroStar Nano. The plate reader was set to read the OD_600 nm_ of each well 30 times in a spiral pattern every half-hour with medium orbital shaking before each read. All media had uninoculated controls whose OD_600 nm_ was subtracted from that of the inoculated medium.

### Technoeconomic analysis

In this study, we used SuperPro Designer v.12 to develop technoeconomic models of HMOs which can be produced from both plant and microbial systems. To do this, we first developed process models and then applied discount cash flow analysis of the theoretical production of LNFPI in plants and microbes. The simplified process flow diagram can be found in Supplementary Fig. 8. In the plant system, we adopted integrated cellulosic biorefinery design to coproduce HMO and ethanol to maximize the use of plant biomass. Biomass sorghum was used as the representative plant because its characteristics, such as high yields and drought tolerance, are ideal for biofuel production. Previous studies demonstrated that using biomass sorghum as the representative plant to accumulate value-added bioproducts could improve the economic performance of an integrated biorefinery^39,40^. Since ethanol is coproduced in the biorefinery, two ethanol selling prices were considered: (1) baseline cellulosic biofuel selling price of US$1.44 l^−1^ of gasoline equivalent and (2) target fuel price of US$1.00 l^−1^ of gasoline equivalent.

Briefly, biomass sorghum with 0.31% dry weight HMOs accumulated in the plant biomass are harvested and transported to the biorefinery gate for preprocessing and short-term onsite storage. HMO extraction, separation and recovery is then conducted on the basis of our laboratory processes and as described in previous texts. Water is used to extract HMO from the biomass (room temperature for 6 h) since the industrial extraction process will last longer than the laboratory-scale process given the large quantity of biomass being processed. The extraction efficiency is assumed at 90% based on previous studies^39^. After extraction, the slurry is first cooled down to room temperature and is sent to centrifugation to remove water. This water is sent to the wastewater treatment unit located in the biorefinery for recycling and reusing. Afterwards, multistage ultrafiltration is used to recover HMO from the extraction stream. Before transporting to HMO onsite storage, another centrifuge is applied to ensure the recovery and purity of the final product. In this plant system, the extracted HMOs are considered as the main product with the purity of >95%. The remaining biomass from biomass sorghum is routed to ionic liquid pretreatment for biomass deconstruction. After ionic liquid pretreatment, enzymatic hydrolysis and ethanol fermentation are conducted to produce ethanol, followed by distillation and molecular sieve to remove excess water. Wastewater from the overall process is routed to the wastewater treatment sector to produce reusable process water and biogas, which can be combusted in the onsite turbogenerator, along with other solids from biomass, to generate heat and electricity that can satisfy the facility’s need. In the microbial system, HMOs were produced as the single product in the biorefinery. Unlike the plant system, pure sugar is used in the microbial system as the sole feedstock and no wastewater treatment sector and onsite combustion are designed for the microbial system. The downstream processing of microbial production is adopted from peer-reviewed publication^41^. After LNFPI is produced in the bioreactor under the bioconversion condition of 30 °C for 52 h, ultrafiltration is first applied to remove biomass and ion exchange chromatography to remove ions and other charged impurities. Multistage nanofiltration is further used to reduce the total volume and remove excess impurities. Gel filtration is used for the final purification of HMO from the microbial production process. The purity of the final product is >98% through this process.

After developing technoeconomic models in SuperPro Designer, we performed mass and energy balance and then applied discounted cash flow analysis to quantify the MSP of HMOs (US$ kg^−1^). For both systems, we assume the biorefinery can operate 24 h per day and 330 days per year for 30 years. The unit price of biomass sorghum is assumed at US$95 per bone-dry tonne and we assume in the plant system the cellulosic biorefinery can intake 2,000 bone-dry tonnes of biomass sorghum per day. The unit price of ionic liquid is US$2 kg^−1^ with a range of US$1–5 kg^−1^. Total capital investment includes installed equipment cost, piping costs, engineering costs, warehouse, site development, construction fees, contingency costs, land costs, startup and working capital. Annual operating costs include raw materials costs, utility costs, labour costs and facility-dependent costs such as insurance. These parameters are kept constant as per the 2011 National Renewable Energy Laboratory report^59^.

### Statistics and reproducibility

No statistical method was used to predetermine sample size as performing experiments in biological triplicate is standard of the field. No data were excluded from the analyses. The experiments were not randomized. The Investigators were not blinded to allocation during experiments and outcome assessment. All experiments looking at individual leaves were conducted in biological triplicate. For the laboratory-scale purifications, extractions were completed in duplicate and each duplicate was measured with two technical replicates. Microbial growth assays were conducted in biological triplicate. For statistical analysis of LNFPI optimization, a heteroscedastic two-tailed Student’s *t*-test with the LNFPI pathway expressed alone was used as the reference group in RStudio v.1.2.5033. GraphPad v.10 was used to plot the microbial growth assays.

### Reporting summary

Further information on research design is available in the Nature Portfolio Reporting Summary linked to this article.

## Supplementary information


Supplementary InformationSupplementary Figs. 1–8 and Tables 1–4 and 6.
Reporting Summary
Supplementary TableSupplementary Table 5.


## Data Availability

The main data supporting the findings of this study are available within the article and its Supplementary Information.
