# Supplementary material for: Engineered plants provide a photosynthetic platform for the production of diverse human milk oligosaccharides
Source: Nat Food. 2024 Jun 13;5(6):480–90. doi: 10.1038/s43016-024-00996-x (PMC11199141; doi:10.1038/s43016-024-00996-x)
Supplement: Supplementary file 1 — Supplementary Figs. 1–8 and Tables 1–6. [file 43016_2024_996_MOESM1_ESM.pdf]

nature food

Supplementary information https://doi.org/10.1038/s43016-024-00996-x

Engineered plants provide a photosynthetic platform for the production of diverse human milk oligosaccharides

In the format provided by the authors and unedited

Supplementary Figure 1. Flow diagram displaying workflows for the characterization and quantification of HMOs from single leaves transiently expressing HMO biosynthetic pathways.

Supplementary Figure 2. Mammalian CMP-Neu5Ac pathway used for production of acidic HMOs.

Supplementary Figure 3. Production of all three HMO classes in a single plant leaf.

Supplementary Figure 4. Effect of overexpression of individual enzymes from the GDP-fucose biosynthetic pathway on HMO profile produced using the LNFPI pathway.

Supplementary Figure 5. RT-qPCR confirms expression of transgenes in stably transformed lines.

Supplementary Figure 6. Representative Extracted Compound Chromatogram (ECC) for *N. benthamiana* oligosaccharides produced from one of the *N. benthamiana* replicates, obtained with an Agilent 6520 NanoChip LC-QToF.

Supplementary Figure 7. Plant-based production improves the economics of LNFPI production.

Supplementary Figure 8. Process models used in this study.

Supplementary Table 1. Identification of HMOs through liquid chromatography-mass spectrometry.

Supplementary Table 2. Gradient of the mobile phase for Triple Quadrupole Liquid Chromatography Mass Spectrometry System (QqQ LC-MS) and MRM transitions for HMOs analysis.

Supplementary Table 3. Concentrations of simple sugars and phenolic compounds following purification of HMOs from plant leaves for use in bacterial growth studies.

Supplementary Table 4. HMO yields following purification for bacterial growth. HMOs were purified from *N. benthamina* transiently expressing the LNFPI and GDP-fucose biosynthetic pathway.

Supplementary Table 6. Primers used in RT-qPCR

Supplementary Figures:

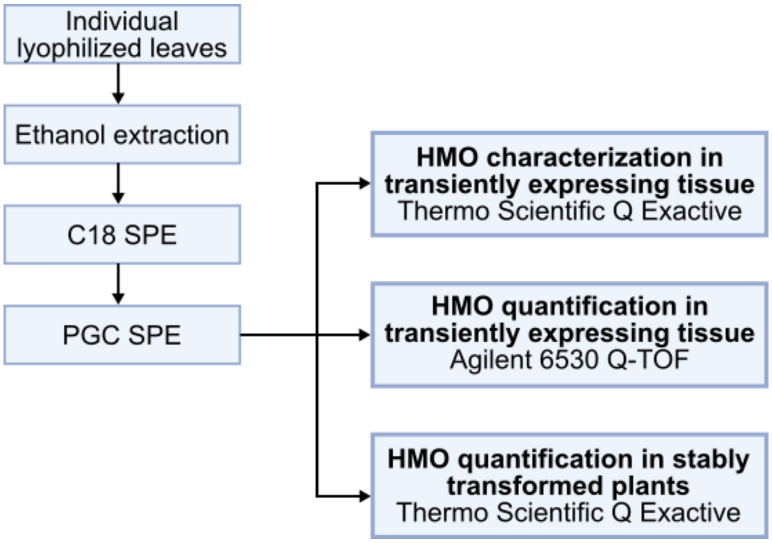

Supplementary Figure 1. Flow diagram displaying workflows for the characterization and quantification of HMOs from single leaves transiently expressing HMO biosynthetic pathways.

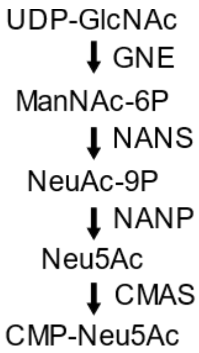

Supplementary Figure 2. Mammalian CMP-Neu5Ac pathway used for production of acidic HMOs.

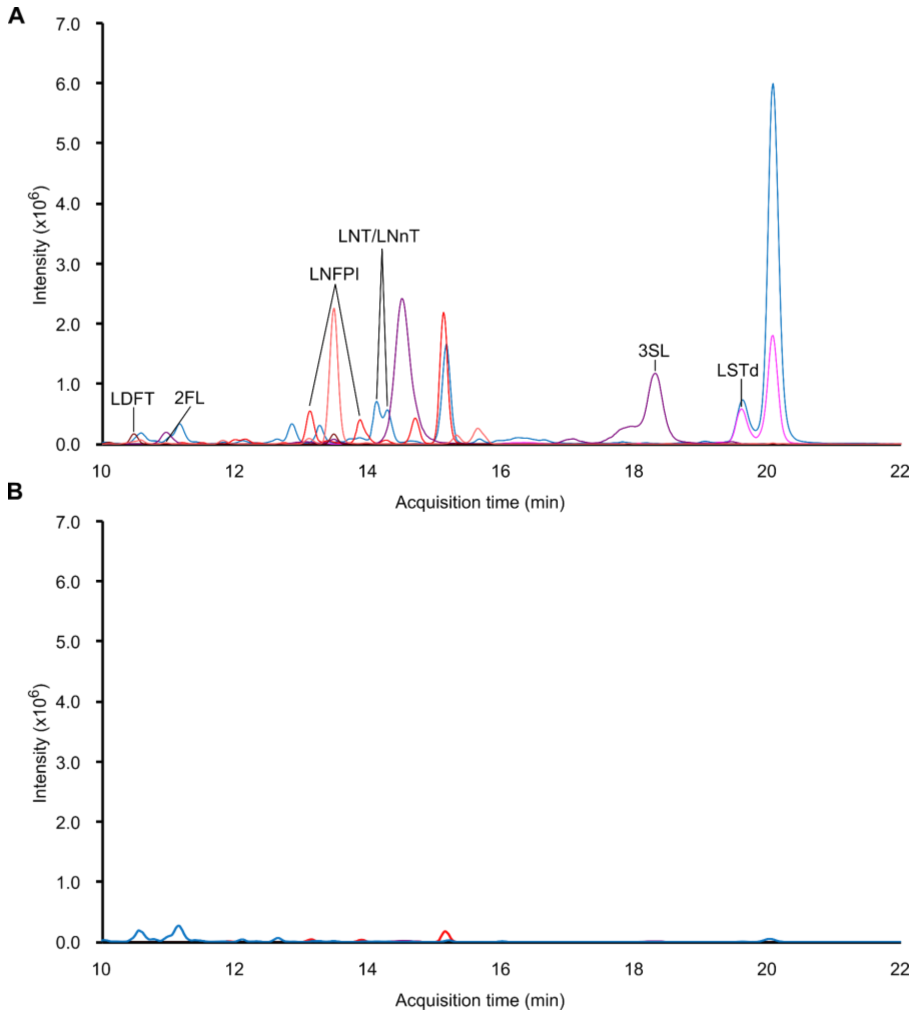

Supplementary Figure 3. Production of all three HMO classes in a single plant leaf. A) Stacked extracted ion chromatogram obtained using Thermo Fisher Scientific Q-Exactive mass spectrometer showing identification of HMOs produced in a single leaf expressing the genes for production of neutral, fucosylated and sialylated HMOs (*GalTpm1141*, *NmLgtA*, *Cvß3GalT*, *Hp0826*, *Te2FT*, *PmSt3*, *St6*, *GNE*, *NANS*, *NANP* and *CMAS*). Additional peaks represent in-source fragments of larger oligosaccharides or additional isomers. B) Stacked extracted ion chromatogram of a single negative control leaf expressing the fluorescent protein, DsRed. Chromatographic separation was performed using a porous graphitic carbon column. All labeled peaks were identified using analytical standards.

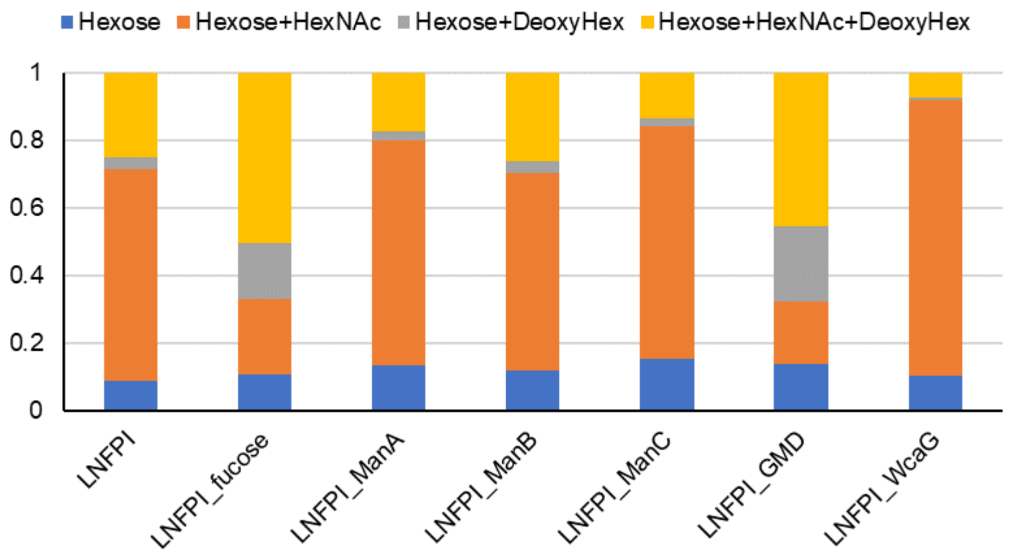

Supplementary Figure 4. Effect of overexpression of individual enzymes from the GDP-fucose biosynthetic pathway on HMO profile produced using the LNFPI pathway. Composition based on Hexose, HexNAc, Deoxyhexose (Deoxyhex) composition determined using *m/z* and MS/MS fragmentation. LNFPI_fucose refers to the overexpression of the LNFPI and full GDP-fucose biosynthetic pathways. Obtained with an Agilent 6530 Accurate-Mass Q-TOF MS.

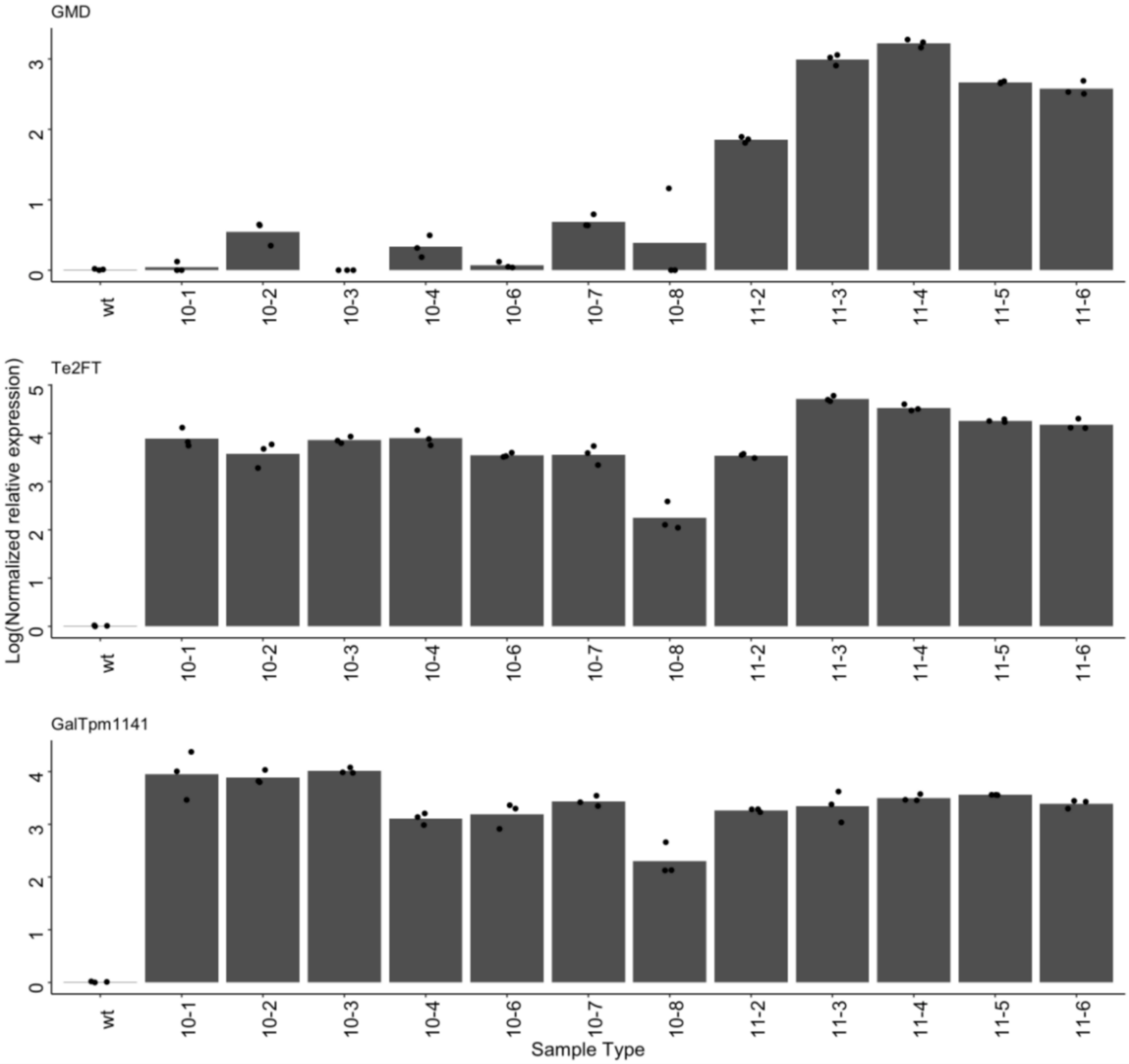

Supplementary Figure 5. RT-qPCR confirms expression of transgenes in stably transformed lines. Expression of three representative genes from each expression cassette (*GMD, Te2FT, and GalTpm1141*) from each transgenic lines confirms expression of transgenes.

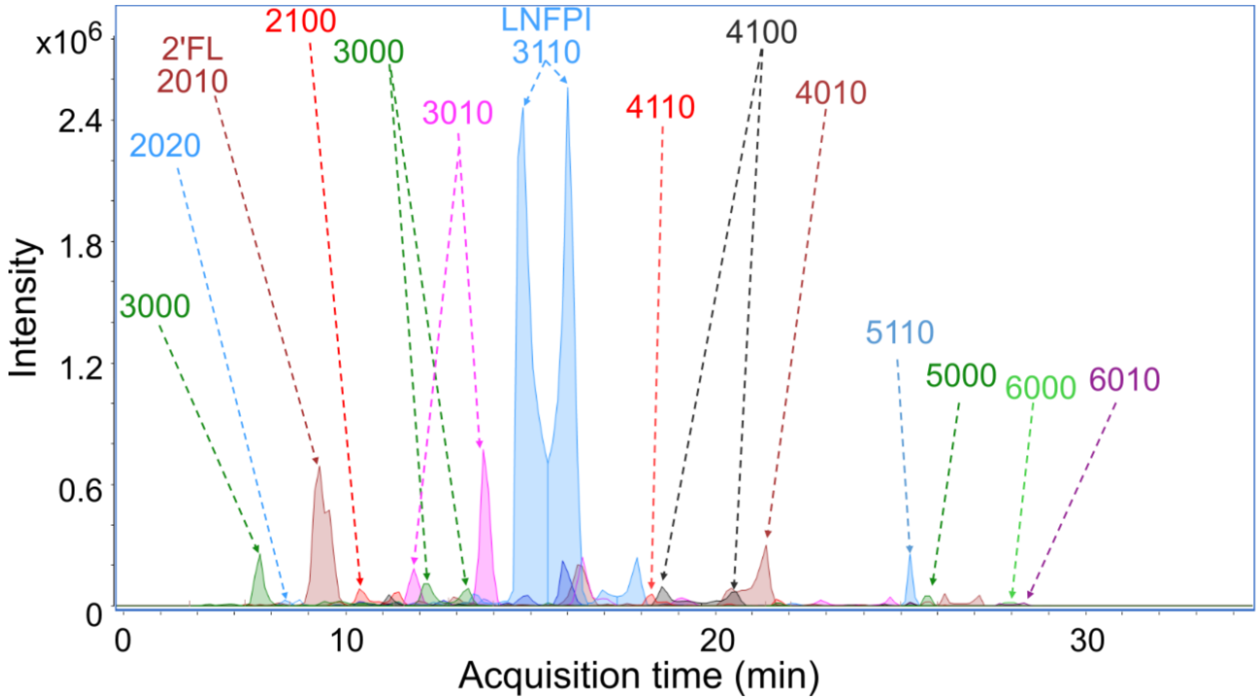

Supplementary Figure 6. Representative Extracted Compound Chromatogram (ECC) for *N. benthamiana* oligosaccharides produced from one of the *N. benthamiana* replicates, obtained with an Agilent 6520 NanoChip LC-QToF. Oligosaccharides names are annotated based on their composition expressed as a numerical in which the first digit represents the number of hexose sugars, second digit indicates the number of hexNAc sugars, third digit indicates the number of deoxyhexose sugars, and the fourth digit represents the number of Neu5Ac.

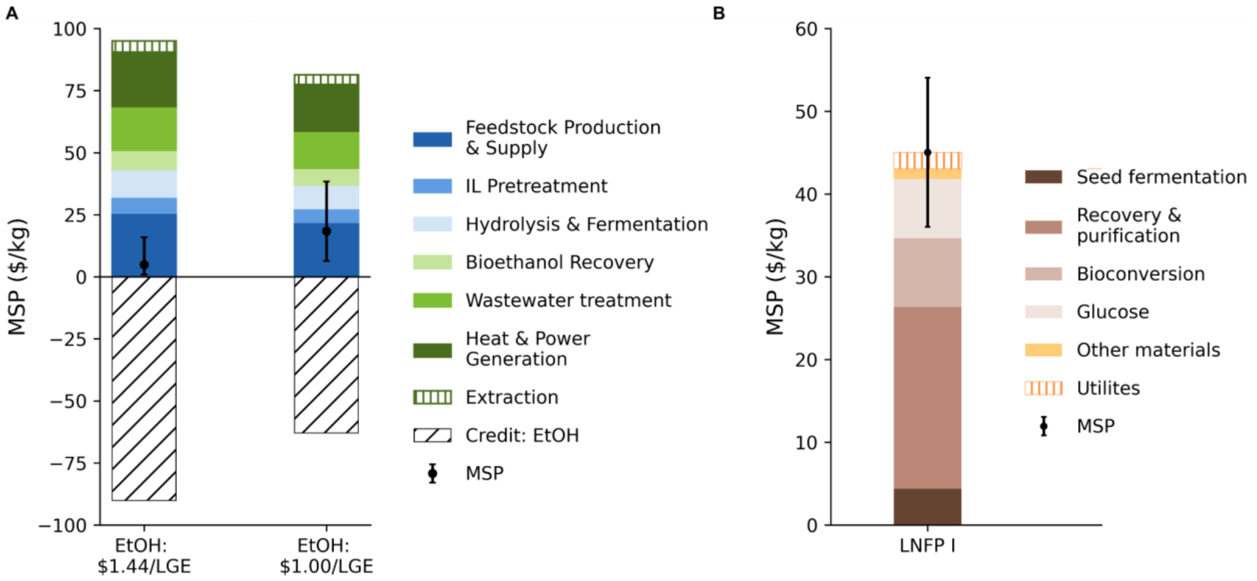

Supplementary Figure 7. Plant-based production improves the economics of LNFPI production. A) Estimated minimum selling price (MSP) of LNFPI produced using biomass sorghum as a model production platform in two bioethanol price scenarios. B) Estimated MSP of microbially produced LNFPI based on inputs, yield, and process described in the highest microbial production of LNFPI[41]. Error bands represent final values calculated with +/- 20% of input parameters.

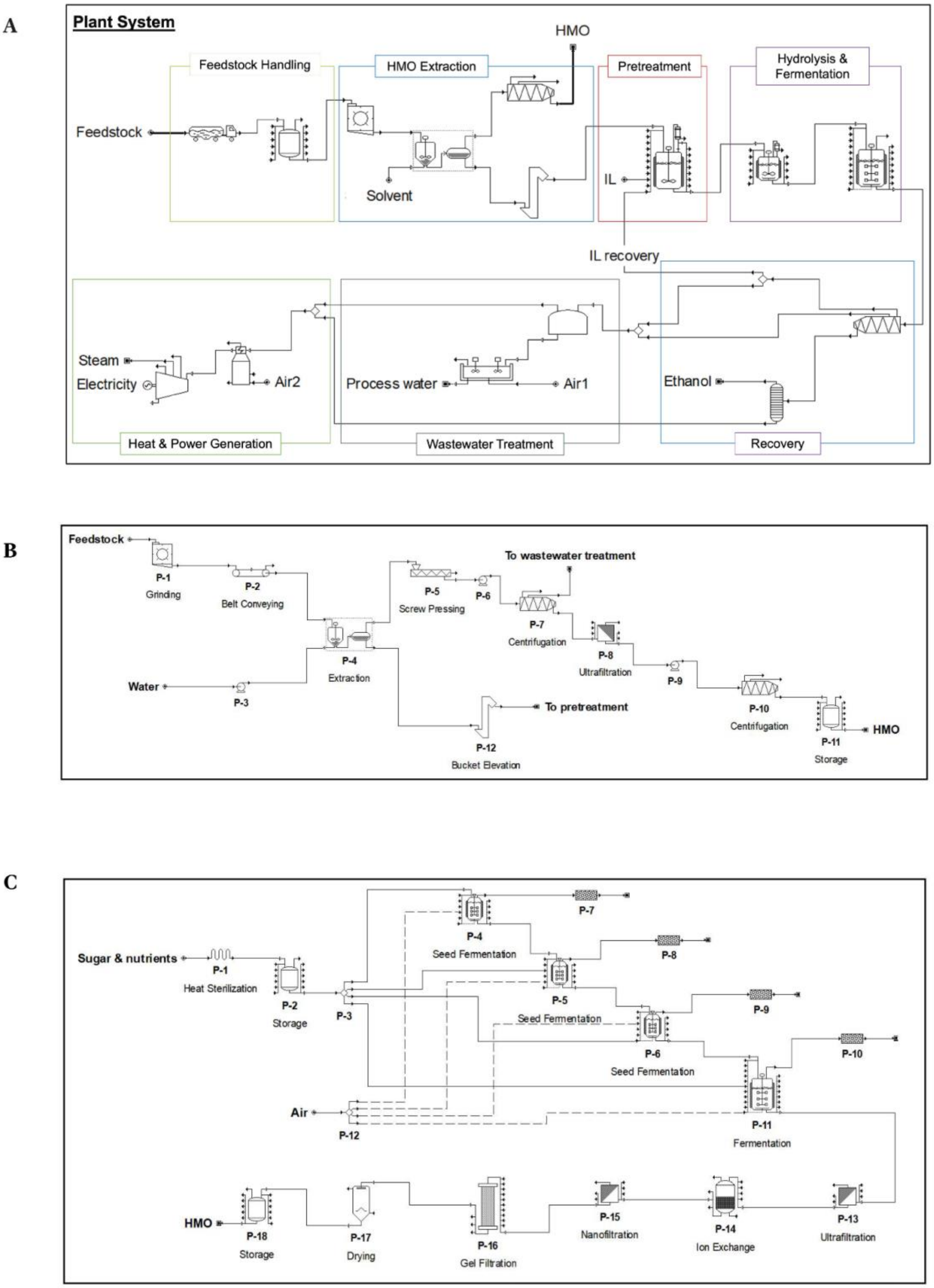
A
Plant System
HMO
Feedstock Handling
HMO Extraction
Pretreatment
Hydrolysis & Fermentation
Feedstock
Solvent
IL
IL recovery
Steam
Electricity
Air2
Process water
Air1
Ethanol
Heat & Power Generation
Wastewater Treatment
Recovery
B
Feedstock
P-1
Grinding
P-2
Belt Conveying
P-5
Screw Pressing
P-6
To wastewater treatment
P-7
Centrifugation
P-8
Ultrafiltration
P-9
P-4
Extraction
Water
P-3
P-10
Centrifugation
P-12
Bucket Elevation
To pretreatment
P-11
Storage
HMO
C
P-7
P-4
Seed Fermentation
P-8
Sugar & nutrients
P-1
Heat Sterilization
P-2
Storage
P-3
P-5
Seed Fermentation
P-9
P-6
Seed Fermentation
P-10
Air
P-12
P-11
Fermentation
HMO
P-18
Storage
P-17
Drying
P-16
Gel Filtration
P-15
Nanofiltration
P-14
Ion Exchange
P-13
Ultrafiltration

Supplementary Figure 8. Process models used in this study. A) Process model of cellulosic biorefinery co-producing biofuel and HMOs. B) Process model of LNFPI extraction process in biomass sorghum. C) Process model of HMO production process in *E. coli*.

Supplementary Tables:

Supplementary Table 1. Identification of HMOs through liquid chromatography-mass spectrometry. Obtained with a Thermo Fisher Scientific Q-Exactive mass spectrometer *denotes HMOs identified with use of authenticated standards.

Name	Hex	HexNAc	Deoxyhex	Neu5Ac	Enzymes	Adduct	*m/z* (experimental)	RT (min)	MS/MS (top 3 by abundance) for peak 1	MS/MS (top 3 by abundance) for peak 2
**2FL***	2	0	1	0	GalTpm1141, NmLgtA, Cvß3GalT, Te2FT, ManA, ManC, Gmd, WcaG	[M+H]+	489.1819	10.99	109.0285, 214.4343, 434.6583	
**3SL***	2	0	0	1	GalTpm1141, NmLgtA, Hp0826, PmSt3, GNE, NANS, NANP, CMAS	[M+H]+	634.2187	18.46	274.0925, 292.1019, 163.0599	
**6SL***	2	0	0	1	GalTpm1141, NmLgtA, Hp0826, St6, GNE, NANS, NANP, CMAS	[M+H]+	634.2191	14.04	Below threshold	
**LDFT***	2	0	2	0	GalTpm1141, NmLgtA, Cvß3GalT, Te2FT, ManA, ManC, Gmd, WcaG	[M+H]+	635.2394	10.53	Below threshold	
**LNT***	3	1	0	0	GalTpm1141, NmLgtA, Cvß3GalT	[M+H]+	708.2559	13.94, 14.46	186.0765, 204.0871, 366.1402	186.0763, 204.0870, 366.1399
**LNnT***	3	1	0	0	GalTpm1141, NmLgtA, Hp0826	[M+H]+	708.2259	14.31	138.0551, 168.0657, 366.1397	
**3_0_0_1**	3	0	0	1	GalTpm1141, NmLgtA, Hp0826, PmSt3, GNE, NANS, NANP, CMAS	[M+H]+	813.2977	14.47	274.0920, 292.1028, 454.1568	
**LNFPI***	3	1	1	0	GalTpm1141, NmLgtA, Cvß3GalT, Te2FT	[M+H]+	854.3136, 854.3137	13.06, 13.80	204.0870, 366.1400, 512.1981	204.0870, 366.1399, 512.1981

**LSTc***	3	1	0	1	GalTpm1141, NmLgtA, Hp0826, St6, GNE, NANS, NANP, CMAS	[M+H]+	999.3505	18.31	Below threshold	
**LSTd***	3	1	0	1	GalTpm1141, NmLgtA, Hp0826, PmSt3, GNE, NANS, NANP, CMAS	[M+H]+	999.351	19.72	204.0871, 366.1398, 657.2349	
**LNDFHI***	3	1	2	0	GalTpm1141, NmLgtA, Cvß3GalT, Te2FT, ManA, ManC, Gmd, WcaG	[M+H]+	1000.3720, 1000.3720	10.27, 10.69	204.0868, 512.1995, 658.2549	
**4_1_0_0a**	4	1	0	0	GalTpm1141, NmLgtA, Cvß3Gal, Hp0826	[M+H]+	870.3079	14.12	204.0869, 366.1398, 708.2563	
**4_1_0_0b**	4	1	0	0	GalTpm1141, NmLgtA, Cvß3Gal, Hp0826	[M+H]+	870.3082	14.97	168.0657, 204.0870, 366.1399	
**4_1_0_0c**	4	1	0	0	GalTpm1141, NmLgtA, Cvß3Gal, Hp0826	[M+H]+	870.3083	15.71	186.0762, 204.087, 366.1398	
**4_1_0_0d**	4	1	0	0	GalTpm1141, NmLgtA, Cvß3Gal, Hp0826	[M+H]+	870.3084	17.17	204.0869, 366.1398, 528.1929	
**4_1_1_0a**	4	1	1	0	GalTpm1141, NmLgtA, Cvß3GalT, Te2FT, ManA, ManC, Gmd, WcaG	[M+H]+	1016.367	14.44	204.0870, 366.1407, 512.1978	
**4_1_1_0b**	4	1	1	0	GalTpm1141, NmLgtA, Cvß3GalT, Te2FT, ManA, ManC, Gmd, WcaG	[M+H]+	1016.366	14.72	204.0869, 366.1395, 512.1978	
**4_1_1_0c**	4	1	1	0	GalTpm1141, NmLgtA, Cvß3GalT, Te2FT, ManA, ManC, Gmd, WcaG	[M+H]+	1016.365	15.16	204.0868, 366.1397, 512.1978	

**4_1_1_0d**	4	1	1	0	GalTpm1141, NmLgtA, Cvß3GalT, Te2FT, ManA, ManC, Gmd, WcaG	[M+H]+	1016.367	16.38	204.0873, 512.1985, 674.2440	
**4_1_1_0e**	4	1	1	0	GalTpm1141, NmLgtA, Cvß3GalT, Te2FT, ManA, ManC, Gmd, WcaG	[M+H]+	1016.366	16.66	204.0869, 366.1398, 512.1980	
**4_1_1_0f**	4	1	1	0	GalTpm1141, NmLgtA, Cvß3GalT, Te2FT, ManA, ManC, Gmd, WcaG	[M+H]+	1016.367	17.67	204.0870, 366.1395, 512.1982	
**4_1_0_1a**	4	1	0	1	GalTpm1141, NmLgtA, Hp0826, PmSt3, GNE, NANS, NANP, CMAS	[M+H]+	1161.404	19.19	292.1021, 366.1401, 657.2355	
**4_1_0_1b**	4	1	0	1	GalTpm1141, NmLgtA, Hp0826, PmSt3, GNE, NANS, NANP, CMAS	[M+H]+	1161.403	20.22	292.1029, 366.1399, 657.2354	
**4_1_0_1c**	4	1	0	1	GalTpm1141, NmLgtA, Hp0826, PmSt3, GNE, NANS, NANP, CMAS	[M+H]+	1161.404	21.39	204.087, 366.1422, 657.2349	
**4_1_0_1d**	4	1	0	1	GalTpm1141, NmLgtA, Hp0826, PmSt3, GNE, NANS, NANP, CMAS	[M+H]+	1161.404	21.74	292.1038, 366.1382, 657.2352	
**4_1_0_1e**	4	1	0	1	GalTpm1141, NmLgtA, Cvß3GalT, PmSt3, GNE, NANS, NANP, CMAS	[M+H]+	1161.404	18.59	204.0872, 292.1036, 657.2360	
**4_1_0_1f**	4	1	0	1	GalTpm1141, NmLgtA, Cvß3GalT, PmSt3, GNE, NANS, NANP, CMAS	[M+H]+	1161.405	20.5	292.1028, 366.1394, 657.2360	

**6_1_0_0a**	6	1	0	0	GalTpm1141, NmLgtA, Cvß3Gal, Hp0826	[M+H]+	1194.414	18.51	366.1401, 690.2460, 1032.3607	
**6_1_0_0b**	6	1	0	0	GalTpm1141, NmLgtA, Cvß3Gal, Hp0826	[M+H]+	1194.414	18.71	366.1399, 528.1936, 690.2488	
**5_2_0_0a**	5	2	0	0	GalTpm1141, NmLgtA, Cvß3Gal, Hp0826	[M+H]+	1235.44	17.93	366.1400, 731.2725, 1235.4442	
**5_2_0_0b**	5	2	0	0	GalTpm1141, NmLgtA, Cvß3Gal, Hp0826	[M+H]+	1235.441	18.13	366.1400, 731.2723, 1235.4406	

Supplementary Table 2. Gradient of the mobile phase for Triple Quadrupole Liquid Chromatography Mass Spectrometry System (QqQ LC-MS) and MRM transitions for HMOs analysis.

Time (min)	A [%]	B [%]	Flow [mL/min]	Max. Pressure Limit [bar]
0.00	13.0	87.0	0.300	600.00
4.00	13.0	87.0	0.300	600.00
5.00	20.0	80.0	0.300	600.00
14.00	28.0	72.0	0.300	600.00
17.00	43.0	57.0	0.300	600.00
18.00	43.0	57.0	0.300	600.00
21.50	65.0	35.0	0.300	600.00
22.50	65.0	35.0	0.300	600.00
24.00	13.0	87.0	0.300	600.00

Compound	Precursor Ion	Product Ion	Fragmentor	Collision Energy	Cell Accelerator Voltage
LNDFH I	1017.4	512.2	180	22	4
LNDFH I	1017.4	204.1	180	42	4
LNFP I	854.3	366.1	175	25	7
LNFP I	854.3	204	175	33	7
2 FL	506.2	163	120	17	7
2 FL	506.2	144.9	120	21	7

Supplementary Table 3. **Concentrations of simple sugars and phenolic compounds following purification of HMOs from plant leaves for use in bacterial growth studies.** Measurements are in mg/g dry weight (dwt) unless otherwise specified. SD indicates standard deviation. Phenolics are measured as mg/g Gallic acid equivalents.

Sample	Glucose	Sucrose	Fuctose	Total	Phenolics
**LNFPI+GDP-fucose pathway (PVPP+C18) for microbial growth**	0.038 ± 0.0028	0.00092 ± 0.000076	0.014 ± 0.0013	0.0533	0.98 ± 0.0054

Supplementary Table 4. HMO yields following purification for bacterial growth. HMOs were purified from *N. benthamina* transiently expressing the LNFPI and GDP-fucose biosynthetic pathway. Measurements are in mg/g dry weight (dwt) unless otherwise specified. SD indicates standard deviation.

Sample	2'FL	LNFPI	LNDFHI	Total HMO	Yield (% dwt)
**LNFPI+GDP-fucose tissue for TEA**	0.69 ± 0.024	3.1 ± 0.038	0.18 ± 0.053	4.5	0.45
**LNFPI+GDP-fucose tissue (PVPP+C18) for microbial growth**	0.38 ± 0.017	1.4 ± 0.16	0.79 ± 0.0059	2.7	0.25

Supplementary Table 6. Primers used in RT-qPCR

Primer Name	Primer Sequence
KM304_EF1a_f	TGAGATGCACCACGAAGCTC
KM305_EF1a_r	CCAACATTGTCACCAGGAAGTG
KM511_GMD_1f	ACCGGGTGATGTGATTATCGCT
KM512_GMD_1r	GGTTTCCAGCCCAGTTTTTCGT
KM517_Te2FT_1f	GTTCTGGAGCATCTGAAGCTGC
KM518_Te2FT_1r	CGGACATCAGCTGCATATCACG
KM523_GalTPM_1f	GCAGAGGACGAGTGGCTATTCT
KM524_GalTPM_1r	AGAAGTCCCTGCCGCAATATGA
