# Supplementary material for: Engineered plants provide a photosynthetic platform for the production of diverse human milk oligosaccharides
Source: Nat Food. 2024 Jun 13;5(6):480–90. doi: 10.1038/s43016-024-00996-x (PMC11199141; doi:10.1038/s43016-024-00996-x)
Supplement: Supplementary file 2 — Reporting Summary [file 43016_2024_996_MOESM2_ESM.pdf]

nature portfolio

Corresponding author(s): Patrick Shih

Last updated by author(s): Mar 22, 2024

Reporting Summary

Nature Portfolio wishes to improve the reproducibility of the work that we publish. This form provides structure for consistency and transparency in reporting. For further information on Nature Portfolio policies, see our Editorial Policies and the Editorial Policy Checklist.

Statistics

For all statistical analyses, confirm that the following items are present in the figure legend, table legend, main text, or Methods section.

n/a	Confirmed	
☐	☒	The exact sample size (n) for each experimental group/condition, given as a discrete number and unit of measurement
☐	☒	A statement on whether measurements were taken from distinct samples or whether the same sample was measured repeatedly
☒	☐	The statistical test(s) used AND whether they are one- or two-sided<br>*Only common tests should be described solely by name; describe more complex techniques in the Methods section.*
☒	☐	A description of all covariates tested
☒	☐	A description of any assumptions or corrections, such as tests of normality and adjustment for multiple comparisons
☒	☐	A full description of the statistical parameters including central tendency (e.g. means) or other basic estimates (e.g. regression coefficient) AND variation (e.g. standard deviation) or associated estimates of uncertainty (e.g. confidence intervals)
☒	☐	For null hypothesis testing, the test statistic (e.g. F, t, r) with confidence intervals, effect sizes, degrees of freedom and P value noted<br>*Give P values as exact values whenever suitable.*
☒	☐	For Bayesian analysis, information on the choice of priors and Markov chain Monte Carlo settings
☒	☐	For hierarchical and complex designs, identification of the appropriate level for tests and full reporting of outcomes
☒	☐	Estimates of effect sizes (e.g. Cohen's d, Pearson's r), indicating how they were calculated

Our web collection on statistics for biologists contains articles on many of the points above.

Software and code

Policy information about availability of computer code

Data collection	Mass spectrometry data was collected using Xcalibur 4.1.31.9 and MassHunter B.07.00. The microplate reader for growth assays was controlled using MARS.
Data analysis	Mass spectrometry data was analyzed using a combination of MS-DIAL 4.90 and MassHunter B.07.00. Technoeconomic analysis was conducted and analyzed using SuperPro Designer v12. Bacterial growth was analyzed GraphPad Prism 10.

For manuscripts utilizing custom algorithms or software that are central to the research but not yet described in published literature, software must be made available to editors and reviewers. We strongly encourage code deposition in a community repository (e.g. GitHub). See the Nature Portfolio guidelines for submitting code & software for further information.

Data

Policy information about availability of data

All manuscripts must include a data availability statement. This statement should provide the following information, where applicable:

- Accession codes, unique identifiers, or web links for publicly available datasets
- A description of any restrictions on data availability
- For clinical datasets or third party data, please ensure that the statement adheres to our policy

Raw mass spectrometry data will be deposited in a public repository.

Human research participants

Policy information about studies involving human research participants and Sex and Gender in Research.

Reporting on sex and gender	n/a
Population characteristics	n/a
Recruitment	n/a
Ethics oversight	n/a

Note that full information on the approval of the study protocol must also be provided in the manuscript.

Field-specific reporting

Please select the one below that is the best fit for your research. If you are not sure, read the appropriate sections before making your selection.

☒ Life sciences ☐ Behavioural & social sciences ☐ Ecological, evolutionary & environmental sciences

For a reference copy of the document with all sections, see nature.com/documents/nr-reporting-summary-flat.pdf

Life sciences study design

All studies must disclose on these points even when the disclosure is negative.

Sample size	No tests were conducted to determine sample size. Sample size was chosen to determine reproducibility based on experience of variability in these assays. For transient expression and bacterial growth experiments, at least three biological replicates were used. For 5 g purifications, extractions were done in duplicate, and each duplicate was measured with two technical replicates. Analysis of stably transformed lines measured metabolite quantity of three leaves on a single transgenic plant. This was the most appropriate way to measure T0 transgenic lines as they only have one plant available.
Data exclusions	No data was excluded for the analysis.
Replication	All transient expression experiments were done in biological triplicate to ensure that different plants still create the same metabolites. Where applicable, these independent measurements were used to generate averages and assess error. All biological replicates showed similar outcomes, indicating the data is reproducible. Furthermore, mass spectral analysis was conducted in multiple labs, with the data being reaffirmed by the analysis conducted in each lab by different personnel. For 5 g purification experiments, two extractions were performed side-by-side, which showed similar results, indicating the data was reproducible. Bacterial growth assays were completed in biological triplicate to ensure data was reproducible between replications.
Randomization	No randomization was done for these experiments. Randomization does not apply because experimental data are measurements of metabolite presence and quantity.
Blinding	There was no blinding as this was not applicable for our experiments. Blinding does not apply because experiment data are measurements of metabolite presence and quantity.

Reporting for specific materials, systems and methods

We require information from authors about some types of materials, experimental systems and methods used in many studies. Here, indicate whether each material, system or method listed is relevant to your study. If you are not sure if a list item applies to your research, read the appropriate section before selecting a response.

Materials & experimental systems

n/a	Involved in the study	
☒	☐	Antibodies
☒	☐	Eukaryotic cell lines
☒	☐	Palaeontology and archaeology
☒	☐	Animals and other organisms
☒	☐	Clinical data
☒	☐	Dual use research of concern

Methods

n/a	Involved in the study	
☒	☐	ChIP-seq
☒	☐	Flow cytometry
☒	☐	MRI-based neuroimaging
